# Decavanadate Salts of Cytosine and Metformin: A Combined Experimental-Theoretical Study of Potential Metallodrugs Against Diabetes and Cancer

**DOI:** 10.3389/fchem.2018.00402

**Published:** 2018-10-02

**Authors:** Eduardo Sánchez-Lara, Samuel Treviño, Brenda L. Sánchez-Gaytán, Enrique Sánchez-Mora, María Eugenia Castro, Francisco J. Meléndez-Bustamante, Miguel A. Méndez-Rojas, Enrique González-Vergara

**Affiliations:** ^1^Centro de Química del Instituto de Ciencias, Benemérita Universidad Autónoma de Puebla, Puebla, Mexico; ^2^Facultad de Ciencias Químicas, Benemérita Universidad Autónoma de Puebla, Puebla, Mexico; ^3^Instituto de Física “Luis Rivera Terrazas”, Benemérita Universidad Autónoma de Puebla, Puebla, Mexico; ^4^Departamento de Ciencias Químico-Biológicas, Universidad de las Américas Puebla, Puebla, Mexico

**Keywords:** decavanadate, metformin, cytosine, X-ray crystal structure, vibrational spectroscopy, ^51^V-NMR, theoretical studies, polyoxovanadates

## Abstract

Cytosine, a DNA and RNA building-block, and Metformin, the most widely prescribed drug for the treatment of Type 2 *Diabetes mellitus* were made to react separately with ammonium or sodium metavanadates in acidic aqueous solutions to obtain two polyoxovanadate salts with a 6:1 ratio of cation-anion. Thus, compounds [HCyt]_6_[V_10_O_28_]·4H_2_O, **1** and [HMetf]_6_[V_10_O_28_]·6H_2_O, **2** (where HCyt = Cytosinium cation, [C_4_H_6_N_3_O]^+^ and HMetf = Metforminium cation, [C_4_H_12_N_5_]^+^) were obtained and characterized by elemental analysis, single crystal X-ray diffraction, vibrational spectroscopy (IR and Raman), solution ^51^V-NMR, thermogravimetric analysis (TGA-DTGA), as well as, theoretical methods. Both compounds crystallized in *P*$\bar{1}\;$space group with *Z'* = 1/2, where the anionic charge of the centrosymmetric ion [V_10_O_28_]^6−^ is balanced by six Cytosinium and six Metforminium counterions, respectively. Compound **1** is stabilized by π-π stacking interactions coming from the aromatic rings of HCyt cations, as denoted by close contacts of 3.63 Å. On the other hand, guanidinium moieties from the non-planar HMetf in Compound **2** interact with decavanadate μ_2_-O atoms *via* N−H···O hydrogen bonds. The vibrational spectroscopic data of both IR and Raman spectra show that the dominant bands in the 1000-450 cm^−1^ range are due to the symmetric and asymmetric ν_(V−O)_ vibrational modes. In solution, ^51^V-NMR experiments of both compounds show that polyoxovanadate species are progressively transformed into the monomeric, dimeric and tetrameric oxovanadates. The thermal stability behavior suggests a similar molecular mechanism regarding the loss of water molecules and the decomposition of the organic counterions. Yet, no changes were observed in the TGA range of 540–580°C due to the stability of the [V_10_O_28_]^6−^ fragment. Dispersion-corrected density functional theory (DFT-D) calculations were carried out to model the compounds in aqueous phase using a polarized continuum model calculation. Optimized structures were obtained and the main non-covalent interactions were characterized. Biological activities of these compounds are also under investigation. The combination of two therapeutic agents opens up a window toward the generation of potential metalopharmaceuticals with new and exciting pharmacological properties.

## Introduction

**Scheme 1:**
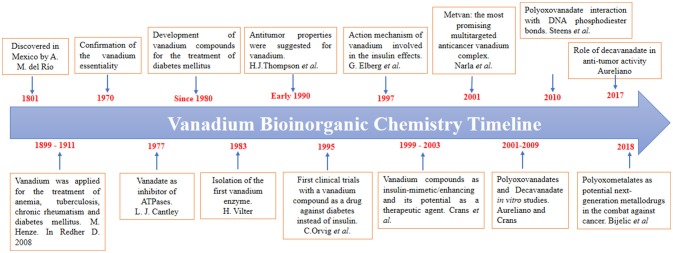
A brief timeline of the vanadium bioinorganic chemistry, highlighting the vanadium role as an antidiabetic, and anticancer agent (Cantley et al., 1977; Thompson et al., 1984; Vilter, 1984; Orvig et al., 1995; Elberg et al., 1997; Crans, 2000; Narla et al., 2001; Rehder, 2008; Aureliano and Crans, 2009; Steens et al., 2010; Aureliano, 2017; Bijelic et al., 2018).

Vanadium bioinorganic chemistry is becoming a very important topic in the last decades. The information about vanadium in biological systems is increasing in giant steps, so as a guide for our research we have considered many highlights in the history of the field (See Scheme 1). In this introduction, relevant information will be presented in a limited way.

Vanadium(IV) and (V) based-compounds have been tested for their potential biomedical use in the treatment of *Diabetes mellitus*, cancer, bacterial diseases and viral infections (Crans, 2000; Rehder, 2008, 2012, 2015a,b). Its therapeutic effects have been attributed mainly to the fact that vanadate has structural and electronic similarities to phosphate. In this sense, vanadium species like vanadate, (H_2_VO_4_)^−^, can adopt a stable trigonal-bipyramidal geometry similar to that of phosphate transition state in phosphate-metabolizing enzymes, and inhibit its biological activity (Costa-Pessoa et al., 2015; Crans, 2015; Dorsey et al., 2018). Since most of the enzymes inhibited by vanadate participate in key intracellular signaling processes, vanadium has been considered a transition element with relevant medicinal applications (Rehder, 2015a,b; Del Carpio et al., 2018; Selman et al., 2018).

Unlike phosphate, vanadate(V) solutions can form, under specific conditions, **polyoxovanadate** species such as the decavanadate anion, [V_10_O_28_]^6−^ (Hayashi, 2011). The structure of this oxo-cluster is stable at acidic pH range and structurally contains ten vanadium atoms assembled into a compact structure with unit cell dimensions of 8.3 Å x 7.7 Å x 5.4 Å, where the V^5+^ metal ions occupy the octahedral interstices in the ten [VO_6_] units (Swallow and Barnes, 1964; Aureliano, 2011).

With respect to the biological activity of decameric species, it has been shown that decavanadate induce several changes in biological processes through the interaction with biological systems, such as myosin, actin, and ion pumps, which are major proteins implicated in muscle contraction and its regulation (Aureliano, 2011, 2016, 2017; Winkler et al., 2017). Furthermore, it has been reported that decavanadate has an insulin-enhancing activity in streptozotocin-induced diabetic rats (Pereira et al., 2009) and in murine diabetic models induced by hypercaloric diets or alloxan (Treviño et al., 2015, 2016, 2018). Recently it has been suggested that it's *in vitro* anticancer activity may be related to the inhibitory effects on specific enzymes involved in tumor proliferation (Aureliano, 2017; Bijelic et al., 2018). Also, the structure determination of the TRMP4 calcium channel has revealed two binding sites for the decavanadate anions, Thus, open up new ways for decavanadate biological actions (Winkler et al., 2017).

**Scheme 2:**
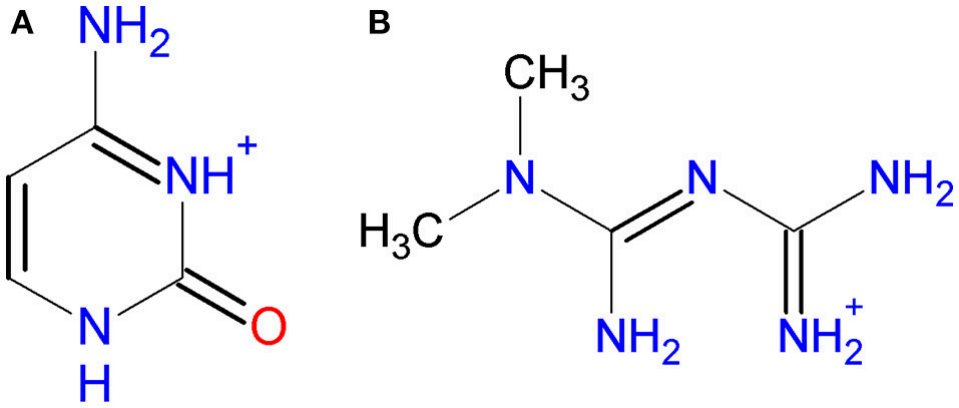
Molecular diagram of **(A)** Cytosinium, HCyt and **(B)** Metforminium, HMetf.

Because of its high anionic charge, decavanadate is mainly stabilized by counterions through electrostatic interactions and **hydrogen bonds**. In previous works, decavanadate has been combined with a variety of cations, including protons, alkali-metal ions, ammonium, alkylammonium, phosphonium, organic and organometallic cations (Chinea et al., 2000; Lee and Joo, 2003; Correia et al., 2004; Kojima et al., 2011; Bartošová et al., 2012; Kioseoglou et al., 2013; Mal et al., 2013; Sánchez-Lombardo et al., 2014; Crans, 2015; Sánchez-Lara et al., 2015, 2016b). In this work, we combined decavanadate with two biologically relevant molecules Cytosine and Metformin (Scheme 2) with the aim to synthesize compounds with potential pharmacological activity.

About the counterions used in this work, Cytosine is one of the naturally occurring nitrogenous bases found in DNA and RNA. Some pyrimidine derivatives exhibit anticancer activity, and some compounds have been used for the treatment of fungal infections (Vermes et al., 2000; Parker, 2009). On the other hand, N,N-dimethylbiguanide or Metformin, as it is known worldwide, is the favorite prescription drug to control the effects of type 2 *D. mellitus* (UK Prospective Diabetes Study (UKPDS) Group., 1998) and is known to affect the cellular housekeeping of copper (Repišcák et al., 2014). Although Metformin is a relatively simple molecule, it possess a versatile behavior as indicated by its capacity of forming salts and coordination compounds in which it participates as a dicationic, monocationic, neutral, and anionic or dianionic species (Zhu et al., 2002).

Besides its role as an insulin sensitizer, new evidence suggests that administration of Metformin may reduce the risk of several types of cancer, including breast, pancreatic and colon cancer (Evans et al., 2005; Libby et al., 2009; Kasznicki et al., 2014). Although the exact molecular mechanism of action for involving Metformin remains unclear, it is known that one of its primary targets is complex I of the mitochondrial electron transport system affecting ATP generation (El-Mir et al., 2000).

Following on from this, the primary focus of this paper is to present two polyoxovanadates-based compounds with potential pharmacological activity. Special emphasis was placed in the compounds' physical and chemical characterization and strengthened with the theoretical analysis. We thus think that the structural study here presented to justify an in-depth biological study to understand, for example, the outcome on the bioactivity of some decavanadate compounds with specific counterions (Yraola et al., 2007; Zorzano et al., 2009; Treviño et al., 2015, 2016, 2018).

## Experimental section

All manipulations were carried out at room temperature and with no special solvent and reagent purification. Infrared spectra were obtained in KBr pellets in the range from 400-4000 cm^−1^ by using an IR Digilab, Mod. Scmitar FT-IR spectrophotometer. Raman spectra were obtained at room temperature in backscattering configuration using the 633 nm line of a He-Ne laser as an excitation source by using a LabRAM HR-Olympus Micro Raman system. The ^1^H, ^13^C were obtained at 500 MHz and 125 MHz respectively, while the ^51^V-NMR spectra were recorded at 131.5 MHz with a Bruker AVANCE III 500 MHz spectrometer using deuterated water (D_2_O). Chemical shifts were referenced to VOCl_3_ as an external standard in the case of ^51^V-NMR. The elemental analysis was performed by Elemental Analyser CHNS/O Thermo Scientific Flash 2000. Thermogravimetric (TGA) and differential thermal (DTA) analysis were carried out under continuous N_2_ flow on an STA 2500 Regulus differential scanning calorimeter (Netzsch Instruments, Burlington, MA). Samples were weighed (~10 mg) and heated from 30 to 700 °C, at a heating rate of 25 °C/min in alumina pans. An empty pan was used as a reference. Single-crystal X-ray data were recorded with an Agilent Gemini A diffractometer, software SHELX-2014/7 (Sheldrick, 2015). Selected crystal data and details of the structure determination of the compounds are summarized in Table 1. CCDC numbers 1850706 (Compound **1**) and 1850707 (Compound **2**) contain the supplementary crystallographic data for this paper. These data can be obtained for free at http://www.ccdc.cam.ac.uk/conts/ retrieving.html (or from the CCDC, 12 Union Road, Cambridge CB2 1EZ, UK; Fax: +44-1223-336-033; e-mail address: deposit@ccdc.cam.ac.uk). The supramolecular networks were studied by using *Mercury* CSD (release 3.1.2) (Macrae et al., 2008), which, together with *OLEX-2* (Dolomanov et al., 2009) were used to produce crystallographic artwork.

**Table 1:** Single crystal data and structure refinement details for [HCyt]_6_[V_10_O_28_]·4H_2_O, **1**, and [HMetf]_6_[V_10_O_28_]·6H_2_O, **2**.

	**Compound 1**	**Compound 2**
Empirical formula	C_24_H_44_N_18_O_38_V_10_	C_24_H_84_N_30_O_34_V_10_
Formula weight	1702.17	1846.61
Crystal system	Triclinic	Triclinic
T (K)	298(2)	298(2)
Space group	*P*$\bar{1}$	*P*$\bar{1}$
*a* [Å]	10.6062(6)	11.5615(6)
*b* [Å]	12.2173(7)	13.2793(8)
c [Å]	12.4329(7)	13.7694(6)
α (deg)	62.225(6)	96.011(4)
β (deg)	69.091(6)	107.347(4)
γ (deg)	85.817(5)	115.557(6)
V (Å^3^)	1323.04(16)	1752.30(18)
Z	1	1
Radiation type	Mo *K*α, λ = 0.7107 Å	Mo *K*α, λ = 0.7107 Å
*D*_calc._ (mg/m^3^)	2.136	1.824
μ(mm^−1^)	1.806	1.37
Reflections collected	18,005	23,664
Independent reflections	6,464	8,617
Parameters	454	567
Goodness-of-fit on *F*^2^	1.03	1.05
Final R index [*I* > 2σ(*I*)]	0.050	0.051
Largest diff. peak and hole (e/Å^3^)	0.80, −0.83	1.01, −0.52
*wR*_2_ (all data)	0.138	0.163

### Computational methods

The structural and electronic properties of compounds [HCyt]_6_[V_10_O_28_]·4H_2_O, **1**, and [HMet]_6_[V_10_O_28_]·6H_2_O, **2**, were computed from theoretical calculations based on the density functional theory (DFT) (Hohenberg and Kohn, 1964). Geometry optimization of the asymmetric unit of Compound **1** and **2** was obtained using the pure functional B97-D3 (Grimme et al., 2011). B97-D3 is a Grimme's functional that includes the Becke and Johnson dispersion corrections (BJ-damping), recommended for non-covalent interactions (Grimme et al., 2011). The split-valence 6-31G(d) basis set (Rassolov et al., 1998) used in the C, H, O, and N atoms includes a single set of Gaussian polarization functions. A LanL2MB basis set (Hay and Wadt, 1985) and an effective core potential (ECP) which replaces the effects of the inner core electrons with a pseudopotential specific for transitions metal atoms, were used for the V atom. Aqueous solvent effects were calculated with the polarized continuum model using the conductor-like polarizable continuum model (CPCM) (Barone et al., 1998). Calculations were performed with the Gaussian16 program (Frisch et al., 2016) and visualization of the results was carried out with the GaussianView 6.0.16 program (Dennington et al., 2016).

### Synthesis of [HCyt]_6_[V_10_O_28_]·4H_2_O (1)

First, 0.5 g of NH_4_VO_3_ was dissolved in 30 mL of distilled water and heated up to dissolution. Then, three drops of concentrated hydrochloric acid (37 %) were added at room temperature to allow the formation of decavanadate anions. After obtaining an orange solution, 0.111 g (1 mmol) of cytosine previously dissolved in 20 mL of distilled water was added dropwise under stirring, obtaining a crystalline orange precipitate, which was filtered from the mother solution (pH = 5.8). Recrystallization from 20 mL of hot water gave a low yield of block-shaped orange crystals, which have low solubility in water. ^51^V NMR (D_2_O): −515.84, −500.31, −423.55. IR (KBr, cm^−1^): 3383, 3321 [*v*_as_(N–H)], 3170 [*v*_s_(N–H)], 2984 [*v*(C–H)], 1718 [*v*(C = O)], 1686 [*v*(C = C)], 1656 [*v*(C = N)], 1545 [δ(N–H)], 987, 952 [*v*(V = O)], 820, 737 [*v*_as_(V–O–V)], 603, 571[*v*_as_(V–O–V)]. Anal. Calc. for C_24_H_44_N_18_O_38_V_10_ (MW = 1702.17 g/mol) C, 16.93%; H, 2.60%; N, 14.81%. Found: C, 17.23%; H, 2.76%; N, 14.94%.

### Synthesis of [HMetf]_6_[V_10_O_28_]·6H_2_O (2)

Two 850-mg-Metformin hydrochloride tablets (Alpharma laboratories) were each crushed using a mortar and pestle and added to a solution containing 0.5 g of NaVO_3_, previously dissolved in 30 mL of distilled water. Three drops of concentrated hydrochloric acid (37%) were added by stirring and then filtered. Needle-shaped orange crystals of Compound **2**, were isolated from their mother solution (pH = 6.3) and filtered after 3 days. It is important to note that the pH value was a key factor to obtain this compound since the metformin molecule can act as monocatioinic or dicationic species at different acidic pH values (Chatkon et al., 2014; Sánchez-Lombardo et al., 2014). The slightly acidic medium of the mother solution maintains the monoprotonation of the metformin molecule and eventually the formation of Compound **2**. Second, the monoprotonated metformin is a good hydrogen-bonding donor leading to better solvation in the aqueous medium, therefore, allowing a good solubility of this compound. Yield (based on vanadium): 0.260 g, 35%. ^1^H NMR (D_2_O): δ = 3.027 (s, 6H, CH_3_), ^13^C (*D*_2_*O)*: δ = 37.7 (CH_3_), 158.7 (C–NH_2_), 160.2 (C = ${\text{NH}}_{2}^{+}$). ^51^V (D_2_O): −514.43, −500.02, −422.31. IR (KBr, cm^−1^): $\tilde{\nu }=$ 3502 [*v*(O–H)], 3396–3321 [*v*_as_(N–H)], 3170 [*v*_s_(N–H)], 1624, 1569 [*v*(C = N)], 1481, 1419 [δ(CH_3_)], 1053 [δ(N–H)], 945 [*v*_s_(V = O)], 830, 736 [*v*_as_(V–O–V)], 603, 571[*v*_s_(V–O–V)]. Anal. Calc. for C_24_H_84_N_30_O_34_V_10_ (MW = 1846.61 g/mol) C, 15.61%; H, 4.60%; N, 22.75%. Found: C, 16%; H, 4.5%; N, 22.74%.

## Results and discussion

### Synthetic strategy

Compound [HCyt]_6_[V_10_O_28_]·4H_2_O, 1, was isolated from an acidic mother solution of NH_4_VO_3_ with a pH of around 6, which allowed the protonation of the cytosine molecules and the formation of decavanadate anions. The first attempt to obtain a compound based on [V_10_O_28_]^6−^ units and cytosine was initially explored by our group using sodium metavanadate salt NaVO_3_ as a precursor of polyoxovanadate ions. However, as a result, we obtained a compound previously published, where the sodium ions are present in the crystal structure forming metal-complexes with the cytosine molecules and the [V_10_O_28_]^6−^ ions (Bošnjakovic-Pavlovic et al., 2009). With the purpose of replacing the Na^+^ ions to get Compound **1**, the starting material NaVO_3_ was replaced by NH_4_VO_3_. In this way, the cytosinium cations were incorporated in the resulting compound, without the presence of ${\text{NH}}_{4}^{+}$ ions, and the water molecules were occluded during the crystallization process as lattice solvent.

On the other hand, crystals of [HMetf]_6_[V_10_O_28_]·6H_2_O, **2**, were isolated in good yield (35%) from an aqueous solution of sodium metavanadate NaVO_3_ at pH 6.3. Again, the pH value and the vanadium source were the key factors to obtain this Compound. The synthetic strategy was, first, take into account that Metformin molecule is a moderately strong base with two pKa values of 2.8 and 11.5 (Kathuria et al., 2018) and therefore can act as monocationic or dicationic species at different acidic pH values. Second, we needed to avoid the formation of metforminium(2+) decavanadates salts, previously reported by us (Sánchez-Lombardo et al., 2014). From this, we decided to use a slightly acidic medium in order to maintain the monoprotonation of the Metformin molecule present in the Metformin hydrochloride tablets, and eventually to get the Compound **2**. Is important to mention herein that the solubility of this compound is very high in contrast with the Compound **1**, may be due to protonated form of Metformin (HMetf) is a good hydrogen-bonding donor, leading to better solvation in aqueous medium allows, therefore, a good solubility of this compound. This point is particularly relevant to carry out biological testing.

### Crystal structure description

**Figure 1:**
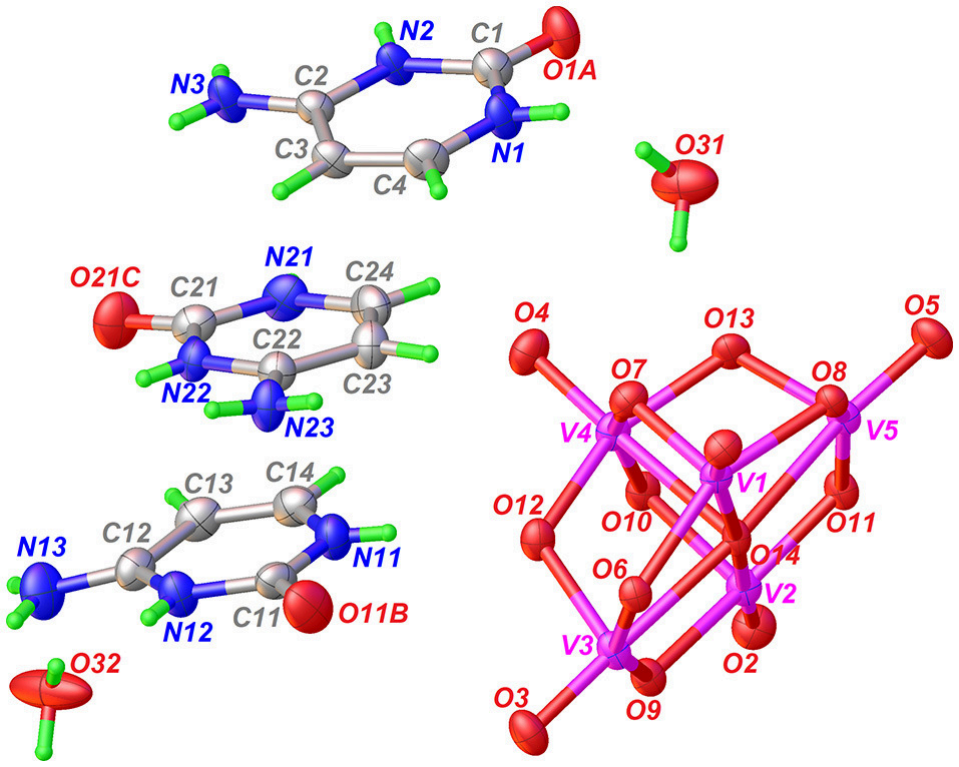
ORTEP-like view of the asymmetric unit of Compound **1**, with displacement ellipsoids for non-H atoms drawn at the 50% probability level.

Single-crystal X-ray structural analysis shows that Compound **1** crystallizes in the centrosymmetric *P*$\bar{1}$ space group (Table 1) with the asymmetric unit, containing one-half of decavanadate anion placed in an inversion center, three independent HCyt cations in the amino-oxo tautomeric form and two lattice water molecules, each located in general positions are depicted in Figure 1. The structure of the [V_10_O_28_]^6−^ cluster is well known and consists of an arrangement of a 10-edge-shared [VO_6_] octahedron close to a local *D*_2*h*_ symmetry, though the crystallographic symmetry is *C*_*i*_.

In agreement with the *Mogul* geometry check (Macrae et al., 2008), the V–O bond lengths and the V–O–V angles are found in normal ranges; comparable with other structures containing this oxo-cluster and showed no unusual geometrical parameters. The backbone of the [V_10_O_28_]^6−^ anion is a very rigid entity that does not present significant structural changes caused by non-covalent interactions. The (3-)anion charge in the asymmetric unit is therefore stabilized by three HCyt cations, protonated at N2, N12, and N22 sites. All the H-atoms of HCyt were found from the difference-Fourier map and refined isotropically. The residual electronic density is actually low for room temperature data of 0.80 e-/Å^3^. From this, Compound **1** can be formulated as [HCyt]_6_[V_10_O_28_]·4H_2_O.

Ionization of the cytosine molecule in an acid medium leads to the introduction of a third donor group. This protonation causes an increase in the internal C–N–C bond angles of the pyrimidine rings in the three independent organic molecules. For example, the C1–N2–C2 angle of 124.81(3)° is larger in cation N1 than in the corresponding value found in the 118.90°-non-protonated cytosine molecule (Lee and Wang, 2010). Also, the short bond lengths between C2–N3 and N1–C4 of 1.302(5) and 1.358(5) Å, respectively, show a resonance effect of π-electron density on the entire molecule. These values are very similar in the three independent cations.

According to the Cambridge Structural Database (CSD, version 5.39, last updated November 2017; Groom et al., 2016), there are no direct structure precedents of Compound **1** in the crystallographic literature, but some complexes containing HCyt or neutral cytosine interacting with polyoxometalate ions have been structurally characterized by X-ray diffraction. One of these structures is an inorganic hybrid polymer built from P_2_Mo_5_O_23_ clusters and Cu(II)–cytosine subunits (Weng et al., 2002). There are two structures based on hetero-polyoxometalates, [SeMo_5_O_21_]^4−^ and [(HAsO4)Mo_6_O_19_]^6−^ (Nagazi and Haddad, 2014; Ayed et al., 2015). The last and possibly the closest structure to Compound **1** is a compound co-crystallized with [V_10_O_28_]^6−^ and Na^+^ ions (Bošnjakovic-Pavlovic et al., 2009). However, a duplex structure containing a cytosine/cytosinium dimer is present in this case. In Compound **1**, dimer formation is disrupted despite a cooperative triple hydrogen bonding of the duplex structure, possibly owing to the low p*K*_a_ value of the hydrochloric acid used to adjust the pH that produces the protonation of all the cytosine molecules, avoiding the formation of the hemicytosinium pair (Perumalla et al., 2013).

The supramolecular structure of Compound **1** is dominated by classic N−H···O, O–H···O, C–H···O and π-stacking interaction among heterocyclic rings. In this regard, the independent cations N1 and N11 of HCyt interact with [V_10_O_28_]^6−^ through N−H···O hydrogen bonds, which involve protonated pyrimidine rings as donor groups and two μ_2_-O sites of the anion as acceptors, with a N1–H1···O9 distance of 2.77(4) Å and a N11–H11···O12 distance of 2.73(4) Å, as well as N–H···O angles of 169 and 172°, respectively (Table 2). These cations generate π-π contacts characterized by a centroid-to-centroid distance of 3.92(3) Å. The remaining N2 and N3 atoms from cation N1 comprise planar rings represented by $R_{2}^{2}\left(8\right)$ (Etter et al., 1990) with μ_2_-O decavanadate atoms.

**Table 2:** Hydrogen bond geometry (Å, °) for Compound **1**.

***D*—H···*A***	***D*—H**	**H*···A***	***D···A***	***D*—H*···A***	**Symmetry code**
N1—H1···O9	0.85 (5)	1.94 (5)	2.777 (4)	169 (5)	*x*−1, *y, z*
N2—H2···O10	0.70 (5)	1.98 (5)	2.670 (4)	168 (5)	–*x*+1, –*y*+1, –*z*+1
N3—H31···O5	0.81 (5)	2.06 (5)	2.813 (4)	156 (5)	*x, y, z*-1
N3—H32···O10	0.81 (5)	2.51 (5)	3.125 (4)	134 (4)	–*x*+1, –*y*+1, –*z*+1
N3—H32···O11	0.81 (5)	2.53 (5)	3.249 (4)	149 (5)	–*x*+1, –*y*+1, –*z*+1
N11—H11···O12	0.74 (5)	2.00 (5)	2.739 (4)	172 (5)	
N12—H12···O32	0.67 (5)	2.01 (5)	2.679 (5)	174 (6)	
N13—H131···O21*C*	0.91 (6)	1.92 (6)	2.818 (5)	168 (5)	–*x*+2, –*y*+1
N21—H21···O31	0.76 (5)	2.16 (5)	2.911 (5)	168 (5)	-*x*+1, -*y*+1, -*z*+1
N22—H22···O11	0.75 (4)	2.03 (5)	2.722 (4)	154 (5)	*x, y, z*-1
N23—H231···O11*B*	0.76 (5)	2.04 (5)	2.791 (5)	173 (5)	-*x*+1, -*y*, -*z*+1
N23—H232···O6	0.81 (5)	1.91 (5)	2.708 (5)	166 (5)	-*x*+1, -*y*, -*z*+1

**Figure 2:**
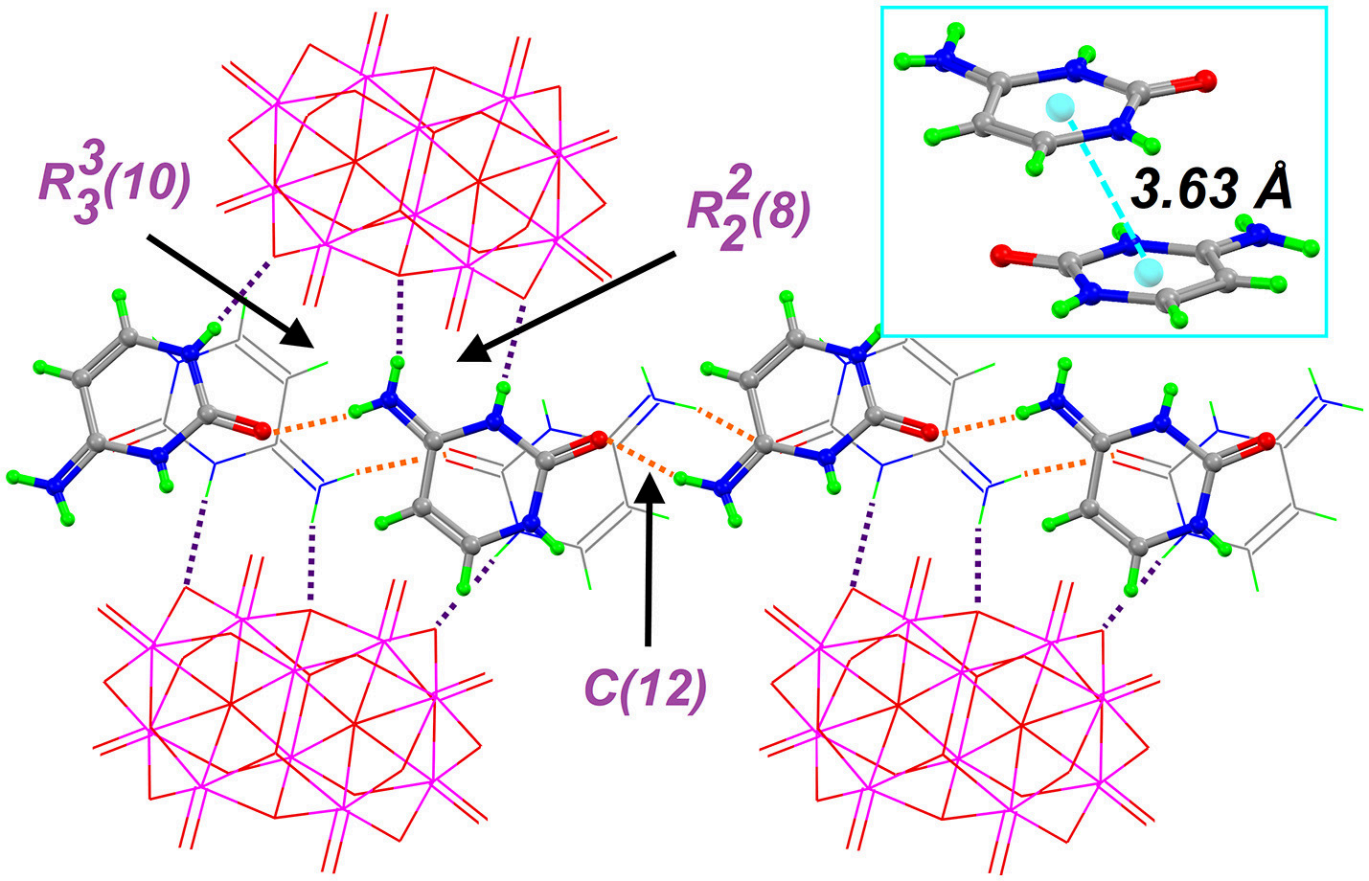
Partial crystal structure of Compound **1**, showing the supramolecular network, which is based on the hydrogen bonds (dashed lines) formed between HCyt and decavanadates: Orange H-bonds are used to form the C(12) chain that is connected to [V_10_O_28_]^6−^ anions by purple H-bonds in the crystal structure. The inset displays the π-π contacts between two non-symmetry related HCyt.

On the other hand, N11- and N21-based HCyt cations interact with each other *via* one hydrogen bond between the acceptor O carboxylic atom and the exocyclic amino group, with distance of 2.81(5) Å for O21C···H131–N13 and 2.79(5) Å for O11B···H231–N23, forming a *C*(12) (Etter et al., 1990) infinite chain extended in the [101] direction, which is stacked through the inversion center coming from the space-group symmetry. In this regard, the π-π parallel-displaced stacking interaction between N11 and N21 cations in the layer is characterized by a centroid-to-centroid distance of 3.63(3) Å and by a displacement angle of approximately 20°. These chains are hydrogen-bonded with decavanadate oxygen atoms forming $R_{2}^{2}\left(8\right)$ and $R_{3}^{3}\left(10\right)$ ring motifs (Figure 2). It is important to mention that distances of 3.6–3.8 Å between centroids of the π-π stacking interactions are also found in DNA and RNA structures. The relevance of this phenomena is unclear at the moment. However, it can be hypothezised that polyoxometalates could be used as templates for base-base linkages and base-base pairing in the early development of life (Rehder, 2011; Cafferty et al., 2016).

**Figure 3:**
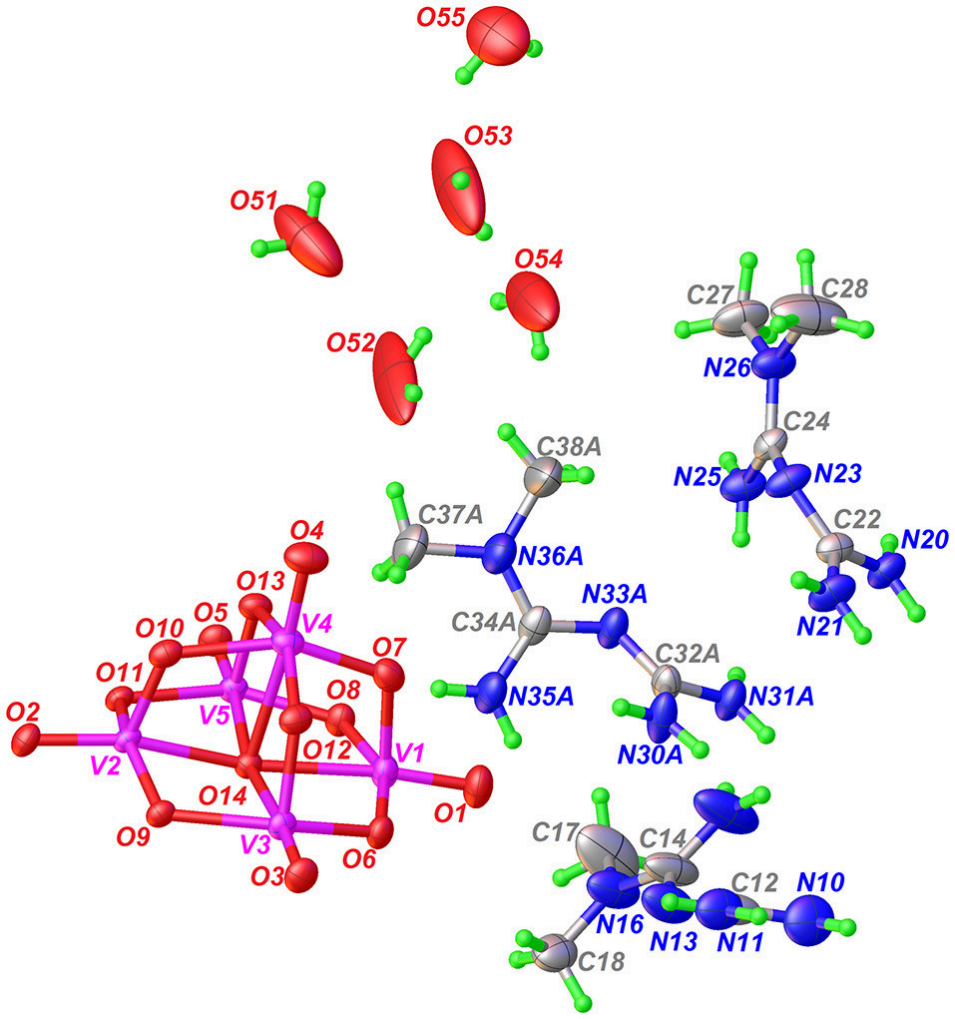
ORTEP-like view of the asymmetric unit of Compound **2**, with displacement ellipsoids for non-H atoms drawn at the 50% probability level. Only the major component of disorder for one HMetf is shown.

The molecular formula of Compound **2** has been determined by single-crystal X-ray diffraction as [HMetf]_6_[V_10_O_28_]·6H_2_O (Table 1). This compound also crystallizes in the triclinic *P*$\bar{1}$ space group with half of the decavanadate anion (*Z'* = $\frac{1}{2}$), three independent monoprotonated HMetf cations and three lattice water molecules in the asymmetric unit (Figure 3). Therefore, six HMetf neutralize the [V_10_O_28_]^6−^ anion charge in the unit cell.

In the refinement process, one of the HMetf is split into two sites with refined occupancies of 0.616(5) in part A and 0.382(5) in part B. It is not surprising to detect a positional disorder for this cation because this limited disorder should be a stabilizing factor for the whole crystal structure. On the other hand, O52, O53, O54, and O55 from water molecules were refined more effectively with an occupancy factor of 1/2. The largest peak in the final difference electron density synthesis was 1.01 e-/Å^3^ and the largest hole was 0.52 e-/Å^3^.

The non-planar HMetf in Compound **2** shows the geometrical features of Metformin hydrochloride (Form B) (Childs et al., 2004), where the orientation of the –C(NH_2_) and –N(CH_3_)_2_ groups are found on the opposite side due to a decrease in van der Waals repulsion. The torsion angles for cations N10, N20 and N30A are 158.3(6)°, 137.2(4)°, and 144.7(13)°, respectively. This type of conformation is observed in several Metforminium(1+) based-salts containing chloride, nitrate, acetate, salicylate, squarate, and carbonate (Zhu et al., 2003; Childs et al., 2004; Olar et al., 2010; Pérez-Fernández et al., 2013; Serb et al., 2014; Dong et al., 2015).

**Scheme 3:**
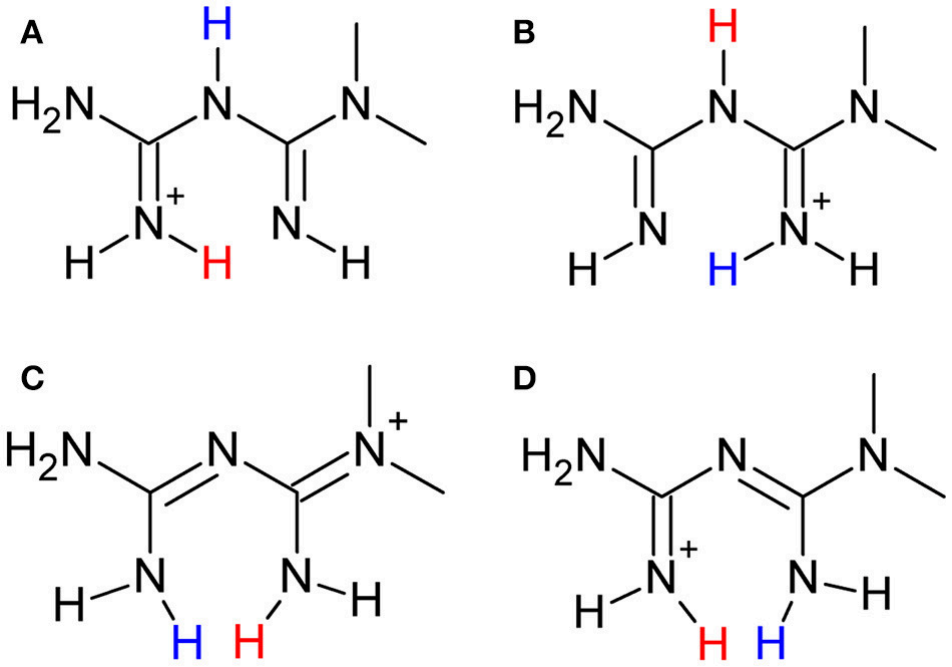
Tautomeric forms **(A–D)** of the metforminium (HMetf) cation.

There are some tautomeric forms reported for HMetf in the solid state, all of which are fully delocalized (Scheme 3) (Pérez-Fernández et al., 2013; Kathuria et al., 2018). Cation N10 present in Compound **2** corresponds to tautomer (c), while N30A and N20 cations correspond to tautomer (d). Although the central N atoms can act as acceptor groups, only N23 accepts one hydrogen bond from O55, with a distance O55–H55A···N23 of 2.84(11) Å (Table 3).

**Table 3:** Hydrogen bond geometry (Å, °) for Compound **2**.

***D*—H···*A***	***D*—H**	**H···*A***	***D*···*A***	***D*—H···*A***	**Symmetry code**
N10—H10*A*···O12	0.86	2.09	2.894 (5)	155	–*x*+1, –*y*+1, –*z*+1
N11—H11A···O7	0.86	2.47	3.251(7)	151	
N11−H11A···O12	0.86	2.37	3.102(6)	143	
N15—H15B···O13	0.85	2.27	2.931	134	
N20—H20A···O5	0.86	2.42	3.142 (5)	142	
N20—H20B···O3	0.86	2.14	2.932(6)	153	
N21—H21*A*···O11	0.86	2.12	2.950(5)	162	
N25—H25*A*···O9	0.86	2.15	2.808(4)	133	–*x*, –*y*+1
N30A—H30*A*···O6	0.86	2.15	2.95(2)	154	
N31A—H31*B*···O6	0.86	2.35	3.09(3)	144	
N35*A*—H35*A*···O8	0.86	2.13	2.898 (6)	149	
O(55)−H55(A)···N23	0.85	2.11(5)	2.841(11)	144(5)	*x*−1, *y, z*

By analyzing the bond lengths in HMetf, we observed that the N–(CH_3_)_2_ methyl group bond lengths correspond to single bonds with a distance ranging from 1.44(7) to 1.47(9) Å. For the C–N bonds, the bond distances range from 1.31(7) to 1.35(7) Å and the central C–N–C angle is reduced to nearly 120°, all of which suggests a π-electron density delocalization across the biguanide group.

Regarding structures crystallized with decavanadate and the anti-diabetic drug Metformin, our research group has previously obtained three decavanadate Metforminium salts at low pH values, all of them characterized by X-ray diffraction (Sánchez-Lombardo et al., 2014). However, in these cases, the Metforminium ions are acting as a dicationic species. Debbie Crans group, on the other hand, has described a water-insoluble double salt containing decavanadate and a mixture of Metforminium(1+) and a protonated guanylurea counterion (Chatkon et al., 2014). The Metformin molecule in the reported compound presents equal proton distributions and similar structural features to those of HMetf found in Compound **2**.

**Figure 4:**
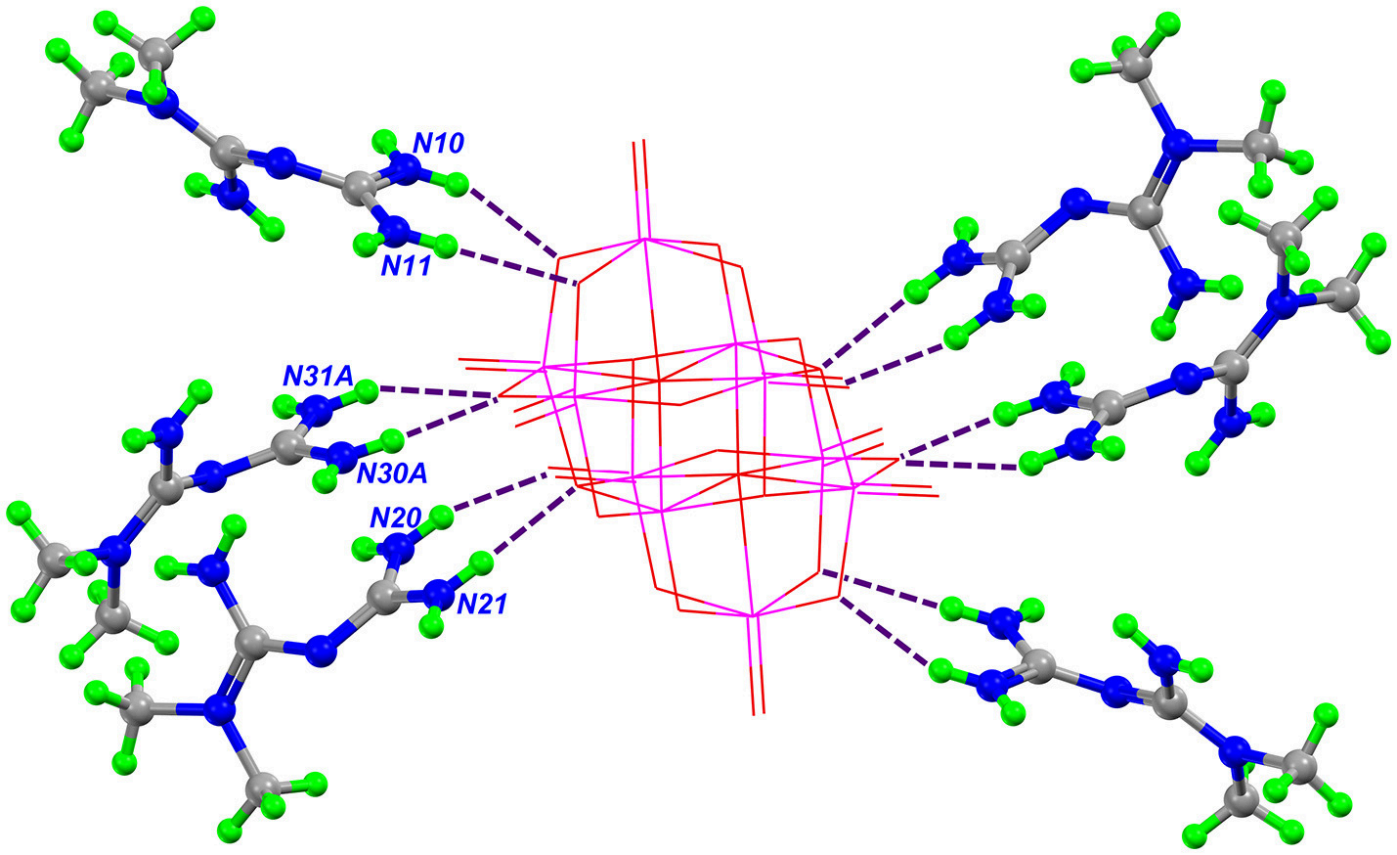
Part of the crystal structure of Compound **2**, showing the interaction between HMetf and [V_10_O_28_]^6−^. The main hydrogen bonds between the cations and anion that form the ${\text{R}}_{2}^{2}$ (8) and ${\text{R}}_{1}^{2}$ (6) rings are shown as purple dashed lines.

Further describing the structure of Compound **2**, all guanidinium moieties of HMetf cations are involved in hydrogen bonds with decavanadate anion, through N–H···O with $R_{1}^{2}\left(6\right)$ and $R_{2}^{2}\left(8\right)$ ring motifs with distances ranging from 2.89(5) to 3.25(7) Å and characterized by angles D–H···A > 140° (Figure 4). These guanidinium-decavanadate interactions are also favored by Coulombic forces.

**Figure 5:**
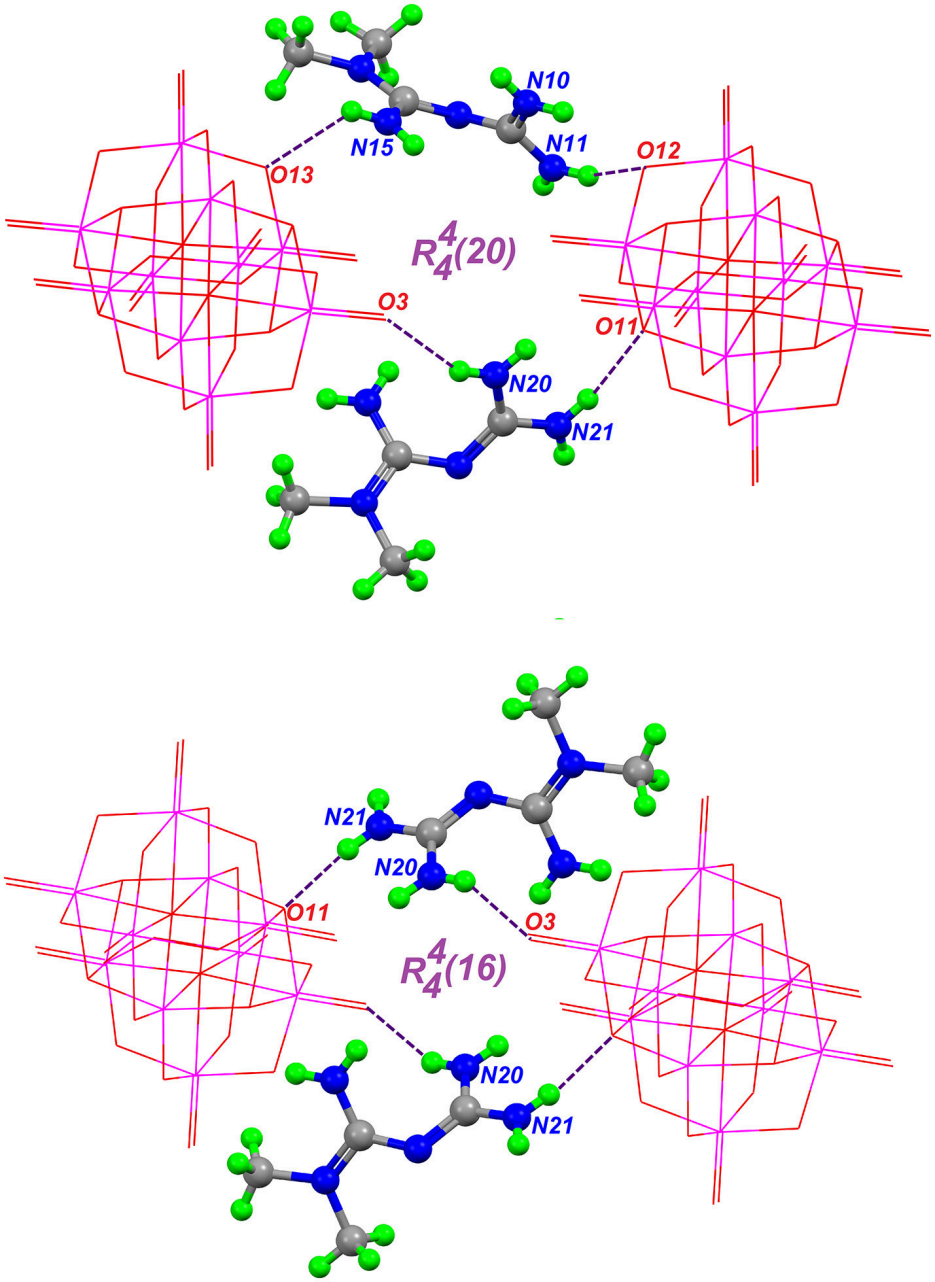
Hydrogen-bond patterns in Compound **2**, formed through hydrogen bonds (purple dashed lines) between two HMetf cations and two symmetry-related [V_10_O_28_]^6−^moities.

A complete supramolecular analysis of this compound is difficult to attain because one HMetf is split into two fractional parts and the disorder in O51, O52, O53, and O54 affect the water H-bond positions. However, considering only the N10 and N20 cations, we observe the formation of a four-level $R_{4}^{4}\left(20\right)$ ring involving two non-symmetry related HMetf, that interact with terminal and bridging μ_2_- and μ_3_-O from [V_10_O_28_]^6−^. This tetramer is formed by N11-H11A···O12 3.10(6) Å, N15-H15B···O13 2.93(8) Å, N20-H20B ⋯ O3 2.93(6) Å, and N21-H21A ⋯ O11 2.95(5) Å hydrogen bonds. Also, a centrosymmetric ring $R_{4}^{4}\left(16\right)$ is present in this compound. In this case, two inversion-related HMetf cations interact with terminal and bridging μ_3_-O atoms from decavanadate as acceptor groups. This third-level motif with pattern ***R***(> k < l > k < l) shows a chair-like conformation. Both supramolecular patterns are represented in Figure 5. It is important to notice the similarity between the binding sites of TRPM4 calcium channel which are rich in arginine and lysine residues and Compound **2**. In this sense, the interaction of the biguanide Metformin, resembles the interactions of the guanidinium ions in the TRMP4 decavanadate binding sites. (see Figure 4) PDB: 5WP6.

### Solid-state vibrational spectroscopy

**Figure 6:**
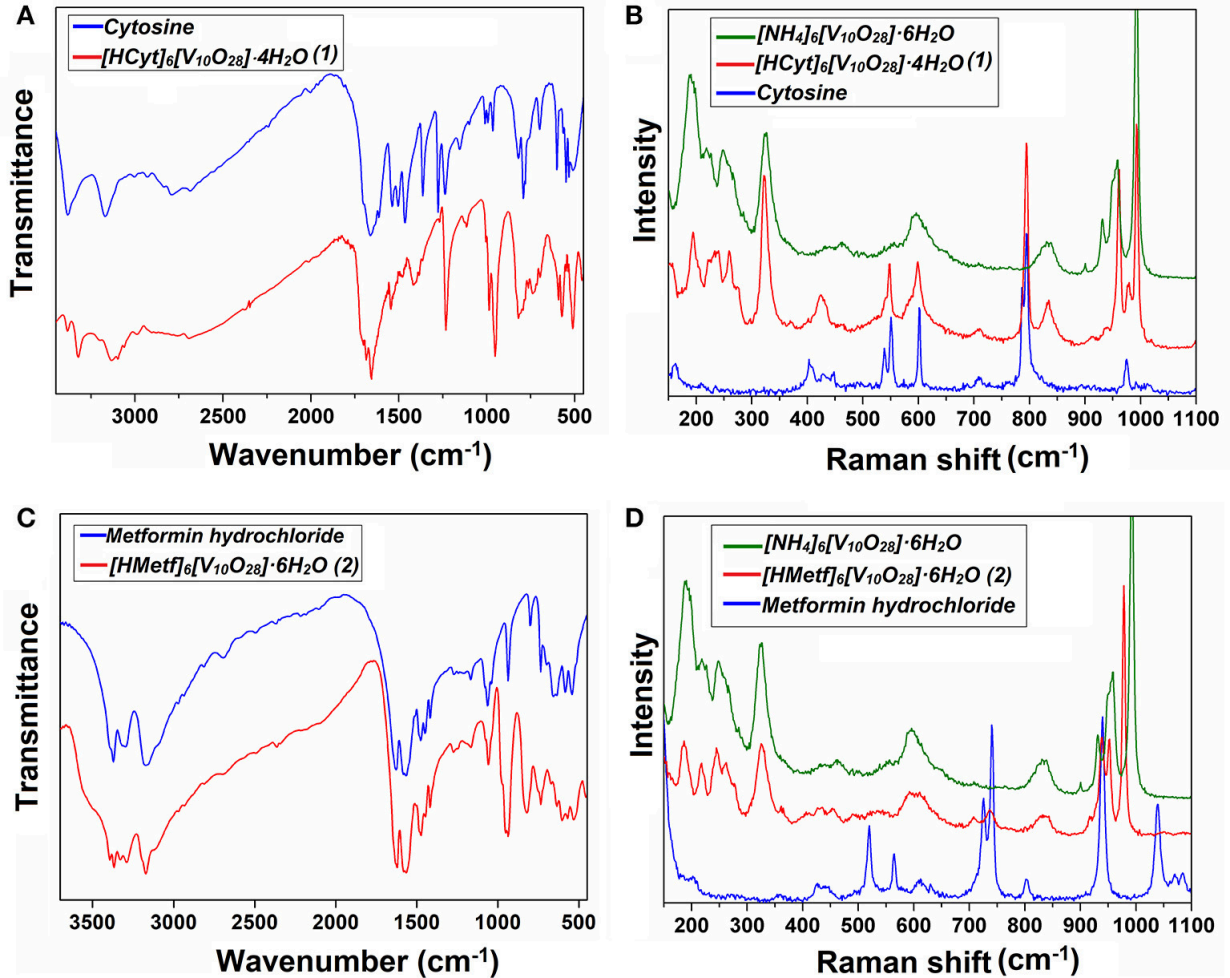
Vibrational spectroscopy of Compounds **1-2** recorded in solid-state. **(A)** and **(B)** are the FT-IR and Raman spectra for Cytosine and Compound **1**, while **(C)** and **(D)** are the FT-IR and Raman spectra for Metformin hydrochloride and Compound **2**, respectively.

FTIR and Raman spectra of Compounds **1-2** are shown in Figure 6 and can be compared with those of neutral cytosine and Metformin hydrochloride, respectively. Furthermore, the Raman spectra of Compounds **1-2** are compared with that of the ammonium decavanadate salt, [NH_4_]_6_[V_10_O_28_]·6H_2_O, for a better assignment of the Raman bands.

The middle IR (Figure 6A) of Compound **1** shows a set of peaks in the *high-frequency* region at 3486-2684 cm^−1^. ν_*as*_(N–H) appears at 3383 and 3321 cm^−1^, while ν_*s*_(N–H) appears at 3135 cm^−1^. *v*(C_*sp*2−−_H) is observed in the range of 2984–2694 cm^−1^ (Rozenberg et al., 2004). Strong IR vibrations seen at 1,718, 1,686, and 1,656 cm^−1^ have been assigned to *v*(C = O), *v*(C = C), and *v*(C = N), respectively. It is known that the position of these bands is shifted upon protonation of the cytosine ring by shortening bond lengths (Rasheed and Ahmad, 2010; Sridhar et al., 2012).

The bands observed at 987 and 952 cm^−1^ in the IR spectra and the corresponding Raman peaks (Figure 6B) at 992 and 958 cm^−1^ are assigned to symmetric stretching modes of the terminal V = O bonds of the decavanadate anion. However, the (V_10_O_28_)^6−^ vibrations are mixed with the corresponding vibrations of the HCyt molecules (see Table 4).

**Table 4:** Assignments of IR and Raman abortion bands for Compounds **1**-**2**.

**Compound 1**			**Compound 2**		
**FT-IR^a^ (cm^−1^)**	**FT-Raman (cm^−1^)**	**Vibrational Assignment^b^**	**FT-IR (cm^−1^)**	**FT-Raman (cm^−1^)**	**Vibrational assignment**
3383 (s)		ν_*as*_(NH_2_)	3532 (b)		ν(O-H)
3321 (s)		ν_*as*_(NH_2_)	3370 (s)		ν_*as*_(NH_2_)
3135 (s)		ν_*s*_(NH_2_)	3201(s), 3171(s)		ν_*s*_(NH_2_)
2693 (vw)		ν(C–H)	3171 (vs)		ν_*s*_(NH_2_)
1718 (vs)		ν(C = O)	2970 (vw)		ν_*as*_(CH_3_)
1686 (vs)		ν(C = C)	2936 (vw)		ν_*s*_(CH_3_)
1656 (s)		ν(N = C)	1624 (s)		ν(C = N)
1545 (m)		ν(N = C), β(Ring)	1569 (vs)		ν(C = N)
1479 (vw)		(C–N)	1509 (vw)		δ(NH)
1231 (s)		ν(C–N), β(C–H), β(N–H)	1477 (m)		δ(CH_3_)
987 (vs), 952 (vs)	992 (vs), 958 (vs)	ν(V = O), γ(C–H), ν(ring), ρ(NH_2_)	1418 (m)		δ(CH_3_)
820 (s), 737 (m)	835 (vs)	ν_*as*_ (V–O–V) + ν(Ring)	1279 (w), 1165 (w), 1059 (w)	1089 (w), 1048 (w)	ν(C–N)
	793 (vs)	ν(Ring)	950 (vs)	977 (vs), 952 (vs), 937 (vs)	ν(V = O), ω(N–H)
572 (s), 513 (s)	595 (vs)	ν_*s*_(V–O–V) + β(C = O), β(Ring)	822 (vs), 735 (vs)	833 (vs), 738 (w)	ν_*as*_(V–O–V), ω(N–H), γ(N–H)
	421 (s)	δ(VO_3_), γ(Ring)	573 (w), 541 (w)	600 (m)	ν_*s*_ (V–O–V), δ(C–N–C)
	321 (s), 260 (s), 237 (s) 162(w)	δ(V–O–V), Lattice vibrations, ω(NH_2_)		325 (s), 261 (s), 245 (s)	δ(V–O–V) Lattice vibrations

a *(s) Strong; (vs) very strong; (m) medium; (w) weak; (vw) very weak; (br) broad; (vbr) very broad*.

b *(ν) stretching; (ν_s_) sym. stretching; (ν_as_) asym. stretching; (β) in-plane bending; (δ) bending; (ρ) rocking; (ω) wagging; (γ) out-of-plane bending*.

The bands at the 833–737 cm^−1^ region in the infrared spectrum are attributed to the asymmetric stretching modes of (V–O–V) units, overlapped with the cytosine ring “breathing” vibration observed at 792 cm^−1^ in the neutral cytosine molecule. In Raman spectra, the asymmetric stretching mode of (V–O–V) is found at 835 cm^−1^ as a medium-intensity peak, and the intense band at 793 cm^−1^ is due to the ring-breathing mode of the HCyt (Mathlouthi et al., 1986; Frost et al., 2005; Frost and Palmer, 2011; Pavliuk et al., 2014; Madzharova et al., 2016; Sánchez-Lara et al., 2016b).

Two IR intense bands at 572 and 513 cm^−1^, and the corresponding to Raman band at 595 cm^−1^ are observed. These frequencies were assigned to ν_*s*_(V–O–V). The Raman bands at 423–320 cm^−1^ may be attributed to the δ(V–O–V). Bands below 240 cm^−1^ are attributed to V−O bonds and lattice vibrations (Frost et al., 2005; Frost and Palmer, 2011).

For Compound **2**, the medium-intensity bands in the *high-frequency* region in the IR spectra (Figure 6C) at 3,395–3,289 cm^−1^ are attributed to the *v*_*as*_(N–H) bond, while the band corresponding to *v*_*s*_(N–H) is observed in the region of 3,200–3,170 cm^−1^. On the other hand, the very weak bands at 2,972 and 2,936 cm^−1^ correspond to the symmetric and asymmetric stretching vibrations of the methyl groups of HMetf, respectively (Gunasekaran et al., 2006). No significant difference between the IR spectra of Compound (**2)** and Metformin hydrochloride were observed possibly because the protonation state remained unchanged.

For Compound **2**, the strong intensity bands in the IR spectrum at 1,624 and 1,569 cm^−1^ could originate from *v*(C = N). The medium-intensity bands in the IR at 1,481 and 1,419 cm^−1^ could arise from asymmetric deformations of CH_3_ groups (Gunasekaran et al., 2006; Sheela et al., 2010; Ghasemi et al., 2018).

The strong IR vibration at 950 cm^−1^ corresponds to *v*(V = O). The corresponding vibrations appear in the Raman spectrum (Figure 6D) at 977 and 952 cm^−1^. In the same Raman spectrum, an intense peak is observed at 938 cm^−1^, which can be assigned to δ(N–H) with a contribution of *v*_*as*_(V–O–V) (Frost et al., 2005; Gunasekaran et al., 2006). The IR absorption at 830 and 736 cm^−1^ corresponds to the *v*_*as*_(V–O–V) bond. The Raman line at 833 cm^−1^ originated from the same vibration is very weak. Returning to the IR absorption spectrum, medium-intensity bands are observed at 603 and 571 cm^−1^, which correspond to *v*_*s*_(V–O–V). This band is observed also at 602 cm^−1^ in the Raman spectrum (Frost et al., 2005; Frost and Palmer, 2011; Pavliuk et al., 2014; Sánchez-Lara et al., 2016b). Vibrations below 450 cm^−1^ are quite similar to those found for Compound **1** crystallized with cytosine.

### Solution NMR-spectroscopy

**Figure 7:**
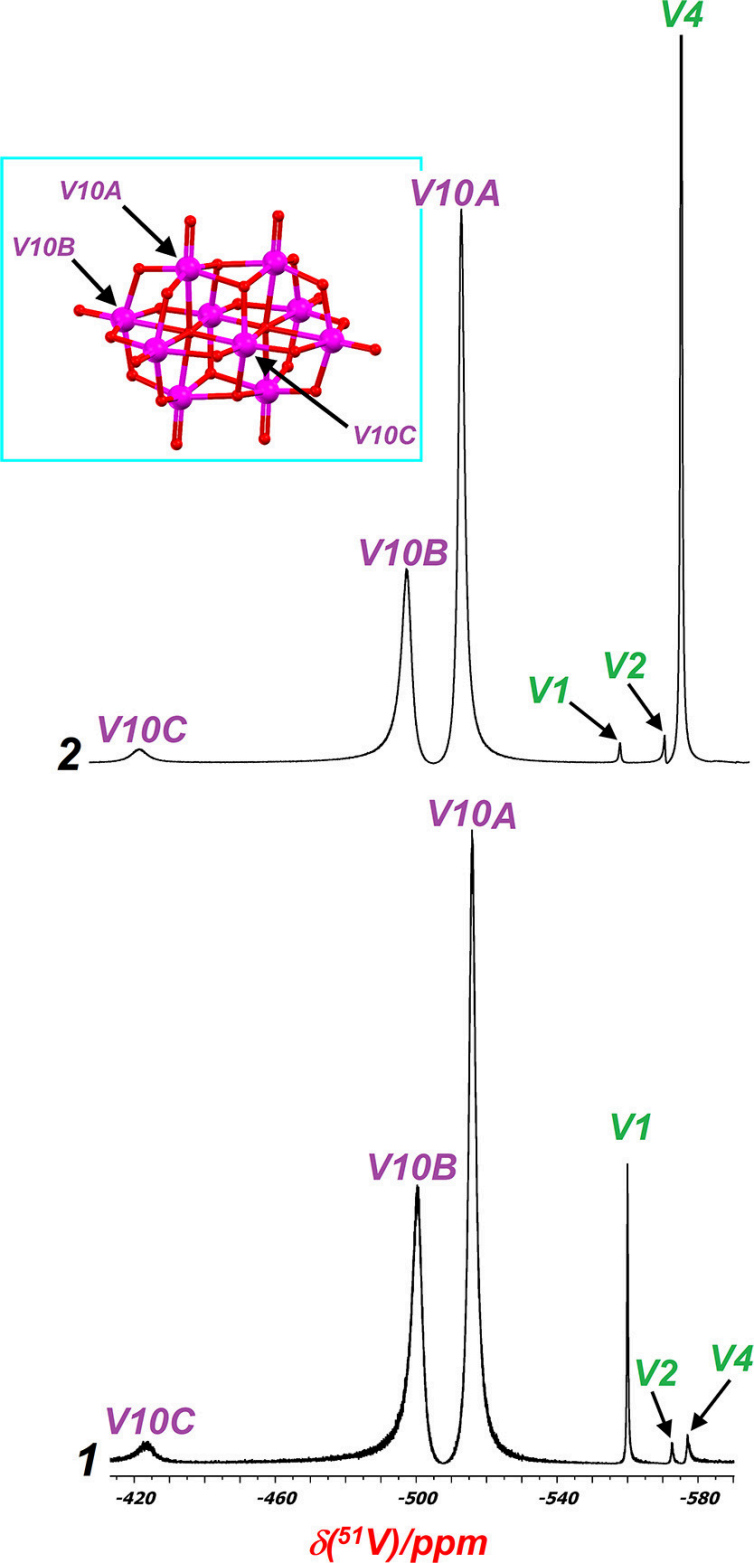
^51^V-NMR spectra in D_2_O of 1-2. The signals correspond to sites VA (low field), VB and VC (high field), and the oligomeric vanadium species V1, V2, and V4. The inset displays the three different vanadium sites found in decavanadate.

It has been shown that vanadate(V) species in solution can occur simultaneously in equilibrium with a different state of protonation and, in some cases, with different conformations (Aureliano and Ohlin, 2014). These characteristics of the aqueous vanadium(V) chemistry are highly dependent on pH, vanadium concentration and ionic strength (Dorsey et al., 2018). Decavanadate structure in solution, for example, could be decomposed into three species: monomeric [H_2_VO_4_]^−^, dimeric [H_2_V_2_O_7_]^2−^ and tetrameric [V_4_O_12_]^4−^ vanadates. This speciation has been studied mainly by ^51^V-NMR spectroscopy and these findings have significant consequences for toxicology activities and pharmacological applications of decavanadate based-compounds (Soares et al., 2007; Aureliano et al., 2016). Due to this last, the ^51^V-NMR spectroscopy for both compounds was carried out. Aqueous solutions of Compound **1** at pH close to 7 display the classical ^51^V resonance signals ascribed to the three different vanadium atoms of the decavanadate structure, present at: V10A = −515.88 ppm, V10B = −500.31 ppm, and V10C = −423.55 ppm (Figure 7) (Rehder et al., 2007; Rehder, 2015a,b). However, it is known that decavanadate slowly decomposes when the pH > 6.5, where it is thermodynamically unstable and it is transformed into the labile monomers and cyclic vanadates (Soares et al., 2007; Aureliano and Crans, 2009). Thus, the sharp signal at δ = −560.04 ppm can be assigned to the biologically active species [H_2_VO_4_]^−^ (V1), while the signals at δ = −572.52 and −577.06 ppm can be attributed to the divanadate [H_2_V_2_O_7_]^2−^ (V2) and cyclic tetramer [V_4_O_12_]^4−^ (V4), respectively (Rehder et al., 2007).

For Compound **2**, the shifts for [V_10_O_28_]^6−^ **structure** are very similar to those for Compound **1**, which are observed at: V10A = −514.43 ppm, V10B = −500, V10C = −422.31 ppm. We also observe the presence of other vanadate species as a consequence of decavanadate hydrolysis, with signals at −560.39, −572.19, and −576.80 ppm, which correspond to V1, V2, and V4, respectively (see Figure 7).

On the other hand, the ^13^C-NMR spectrum (Figure S1) shows one signal at 37.41 ppm corresponding to the equivalent methyl groups of the Metformin molecules, and the two signals observed at 158.43 and 160.12 ppm corresponding to the tertiary guanidyl carbons C–NH_2_ and C = ${\text{NH}}_{2}^{+}$, respectively (Chatkon et al., 2013; Ibrahim et al., 2015). The ^1^H-NMR in water-*D*_2_ shows only one resonance peak at 2.7 ppm due to two equivalent methyl groups (data not shown). The N–H protons were exchanged in D_2_O by eliminating the signals at 7.20 and 7.69 ppm, previously observed in DMSO-*d*_6_ (Gadape and Parikh, 2011).

### DSC-TGA analysis

The thermal behavior of [HCyt]_6_[V_10_O_28_]·4H_2_O **(1)**, and [HMetf]_6_[V_10_O_28_]·6H_2_O **(2**), was assessed by TGA/DTA analysis (Figures S2, S3). For Compound **1**, the thermogram shows a mass loss of around 3-4% corresponding to lattice water molecules in the range of 30–150°C, which fits with four water molecules [% mass, calc. (found): 4.23% (4%)]. An endothermic process occurs around 225°C which may be attributed to the melting of cytosine, followed by a continuous mass loss in the range from 220 to 580°C which may correspond to the thermal degradation of the six cytosinium ions [% mass, calc. (found) for 6 × C_4_H_6_N_3_O: 39.5% (40%)]. For Compound **2**, the DTA thermogram shows three well defined endothermic peaks in a temperature range of 37–70°C, all related to the loss of lattice water molecules. This water loss is in agreement with the corresponding mass change observed in the TGA curve suggesting the loss of six uncoordinated water molecules [% mass, calc (found): 5.85% (6%)]. Completion of this stage leads to the anhydrous phase, which shows a significant range of thermal stability extending to ca. 200°C. This thermal behavior has been observed for other polyoxometalates ions with organic moieties (Iyer et al., 2014; Sánchez-Lombardo et al., 2014; Martin-Caballero et al., 2016; Dissem et al., 2018). Considering that the melting point of Metformin hydrochloride is 224°C (Benmessaoud et al., 2016), we can assign the endothermic peaks observed in the DTA thermogram in a temperature range of 190–210°C, to the melting of Metforminium cations (HMetf) present in Compound **2**. Above this temperature, an exothermic mass loss takes place that corresponds to the combustion of the metforminium cations per decavanadate unit [%mass, calc. (found) for 6 × C_4_H_12_N_5_: 42.3% (40%)]. In both cases, above 540–580°C no further mass losses are observed, which can be attributed to the remaining inorganic fragment [V_10_O_28_]^6−^ which is thermally stable event up to 600°C as observed in previously reported decavanadates compounds (Omri et al., 2015; Ortaboy et al., 2018). It may be possible that further thermal treatment of the decavanadate ion may yield other vanadium oxides, possibly V_2_O_5_ or other mixed-valence oxides (Riou et al., 1998). However, a more in-depth analysis such as thermodiffractometry is necessary to confirm the nature of the resulting metal oxides, which may probably consist of vanadium oxide nanoparticles produced by thermal decomposition of the polyoxovanadate systems (Martin-Caballero et al., 2016).

### Theoretical results

**Figure 8:**
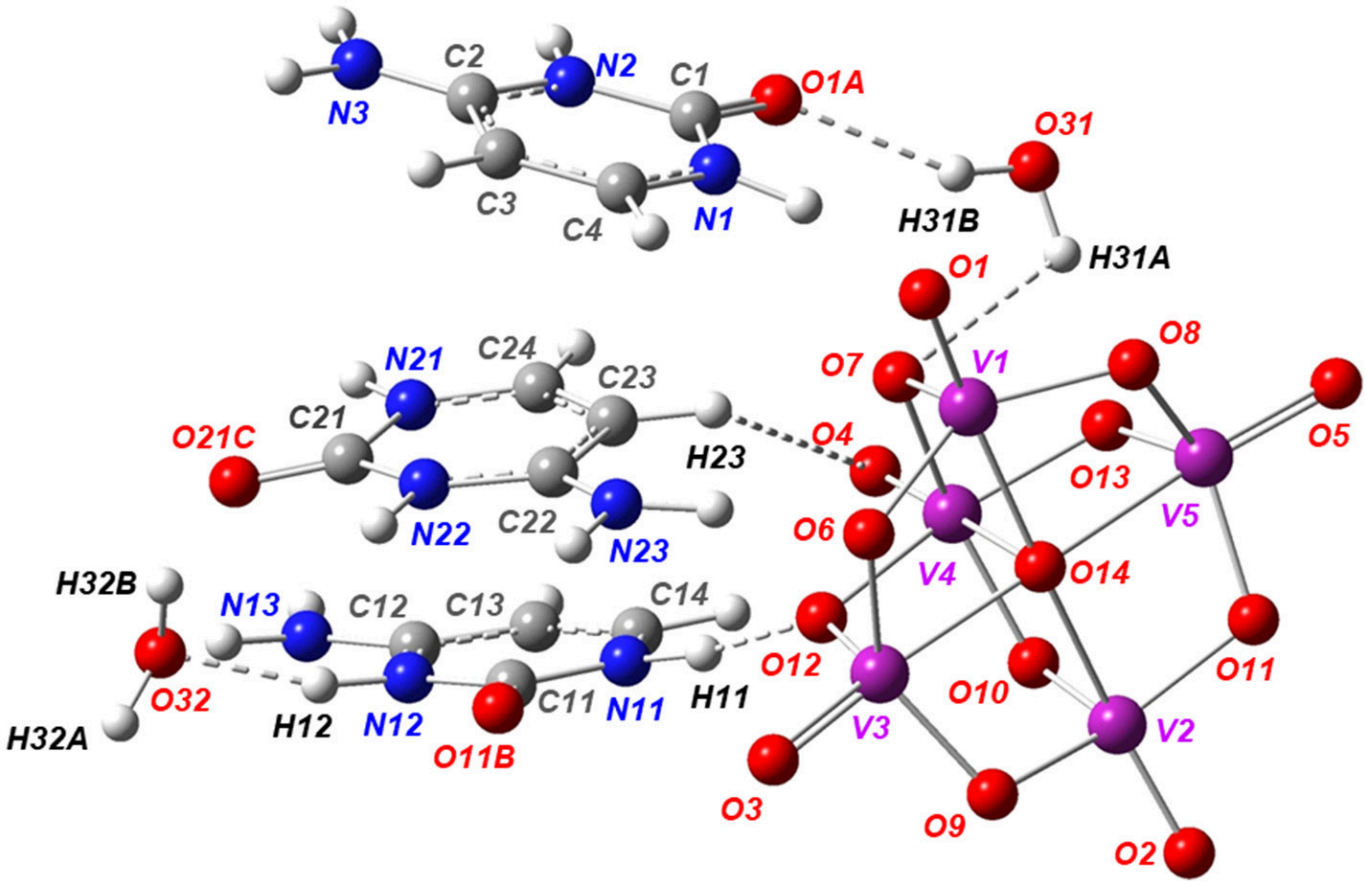
Solution-phase of the optimized structure of the asymmetric unit of Compound **1**, using the B97-D3 functional with the 6-31G(d) basis set for the C, H, O, N, atoms and the LanL2MB basis set for the V atoms.

**Figure 9:**
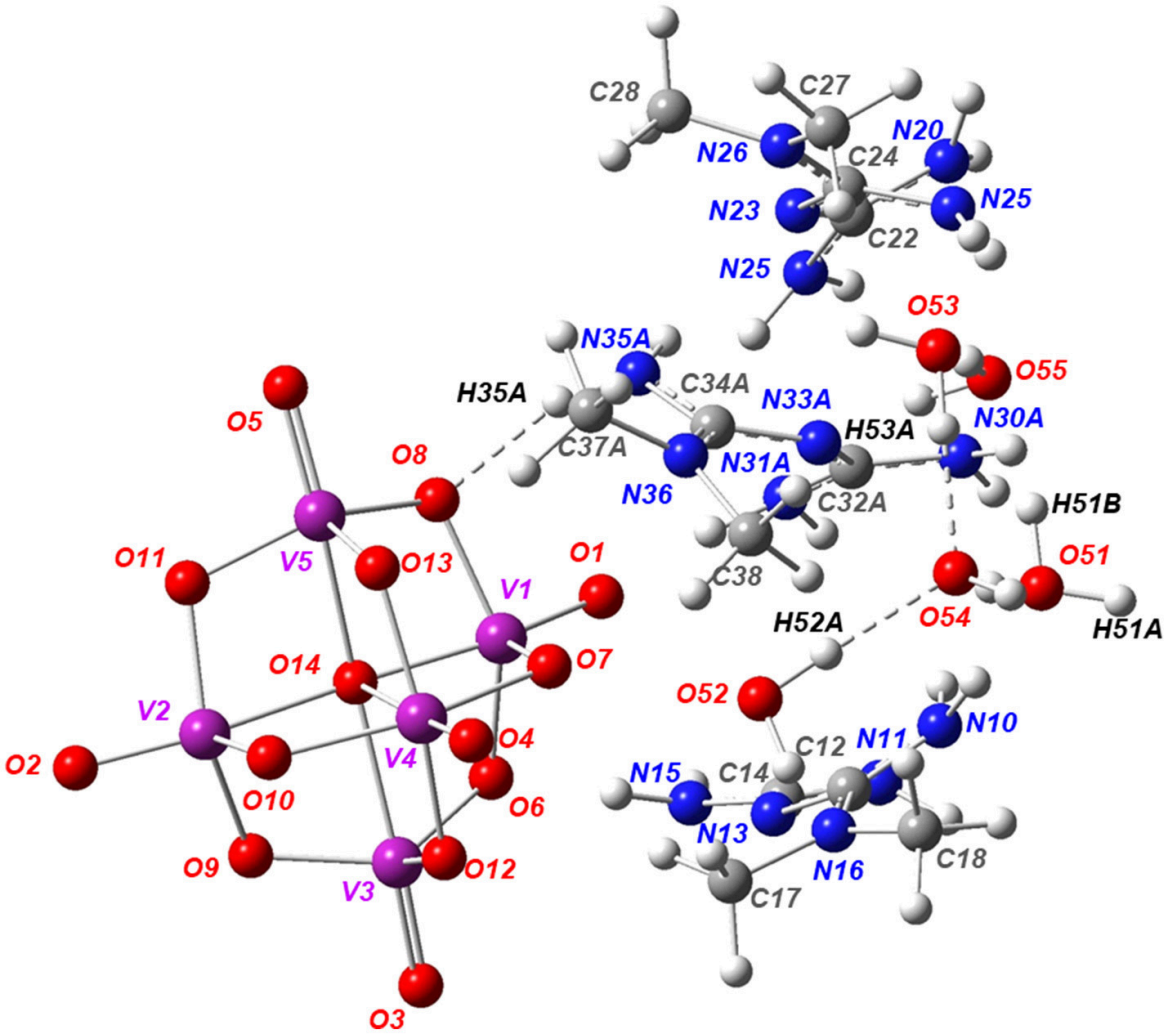
Solution-phase optimized structure of the asymmetric unit of Compound **2** using the B97-D3 functional with the 6-31G(d) basis set for the C, H, O, N, atoms and the LanL2MB basis set for V atoms.

Optimized structures of the asymmetric unit in aqueous solvation phase of Compounds **1** and **2** are shown in Figures 8, 9. It can be seen that the theoretical structures are similar to those mapped from crystallographic data. Selected parameters for the compounds are shown in **Tables S1, S2**. Selected hydrogen bonds are also reported in Table S3. Atom labels are the same as those used in Figures 1, 3.

Table S1 shows the structural parameters for Compound **1** obtained by the DFT-D methods in solution phase from X-ray crystallographic data used as an initial structure for calculation. In the aqueous phase, some variations were observed in the dihedral angle values calculated in the decavanadate anion [V_10_O_28_]^6−^ due to the solvent effect. The group of cytosinium cations ([C_4_H_6_N_3_O]^+^) interacting with the decavanadate anion [V_10_O_28_]^6−^ replicates the behavior in the crystallographic unit cell. Water molecules in Compound **1** form important hydrogen bond interactions.

For Compound **2**, the full optimization of the asymmetric unit showed that the intramolecular parameters for the metforminium cations ([C_4_H_12_N_5_]^+^) had significant structural displacements relative to the position of the decavanadate. The decavanadate ion optimized parameters for Compound **2** were obtained with good precision using the crystallographic data as a reference. Moreover, the optimized parameters for the anion structure were similar in both compounds. Contrary to what is observed in **1**, where one of the water molecules acts as a water bridge (μ_2_-O) between the cation and the decavanadate, the optimized water molecules in Compound **2** act as a cluster (O51–O55) without any apparent cation-anion interaction (Figure 9).

Including the solvent effect in both compounds was a decisive step for geometry optimization, as well as taking into consideration the non-covalent interactions N–H···O and O–H···O through the inclusion of the dispersion forces by the DFT-D methods.

The main hydrogen bonds were also theoretically characterized. A previous study about the electronic properties of a cytosine-decavanadate system was carried out by using Atoms in Molecules Topological Analysis (Bošnjakovic-Pavlovic et al., 2009). In this work, the density of D–H···A hydrogen bonds were analyzed, suggesting the N–H···O as strong hydrogen bonds and C–H···O as weak hydrogen bonds in the cytosine-decavanadate system containing sodium cations. In the present work, the non-covalent interactions N–H···O were also found using the DFT-D methodology and including a solvent effect by means of the CPCM approach. Values similar to crystallographic data, used as a reference, were obtained for interactions N–H···O for Compounds **1** and **2** as reported in Table 2. In addition, important interactions were calculated between water molecules in both compounds (Table S3).

**Figure 10:**
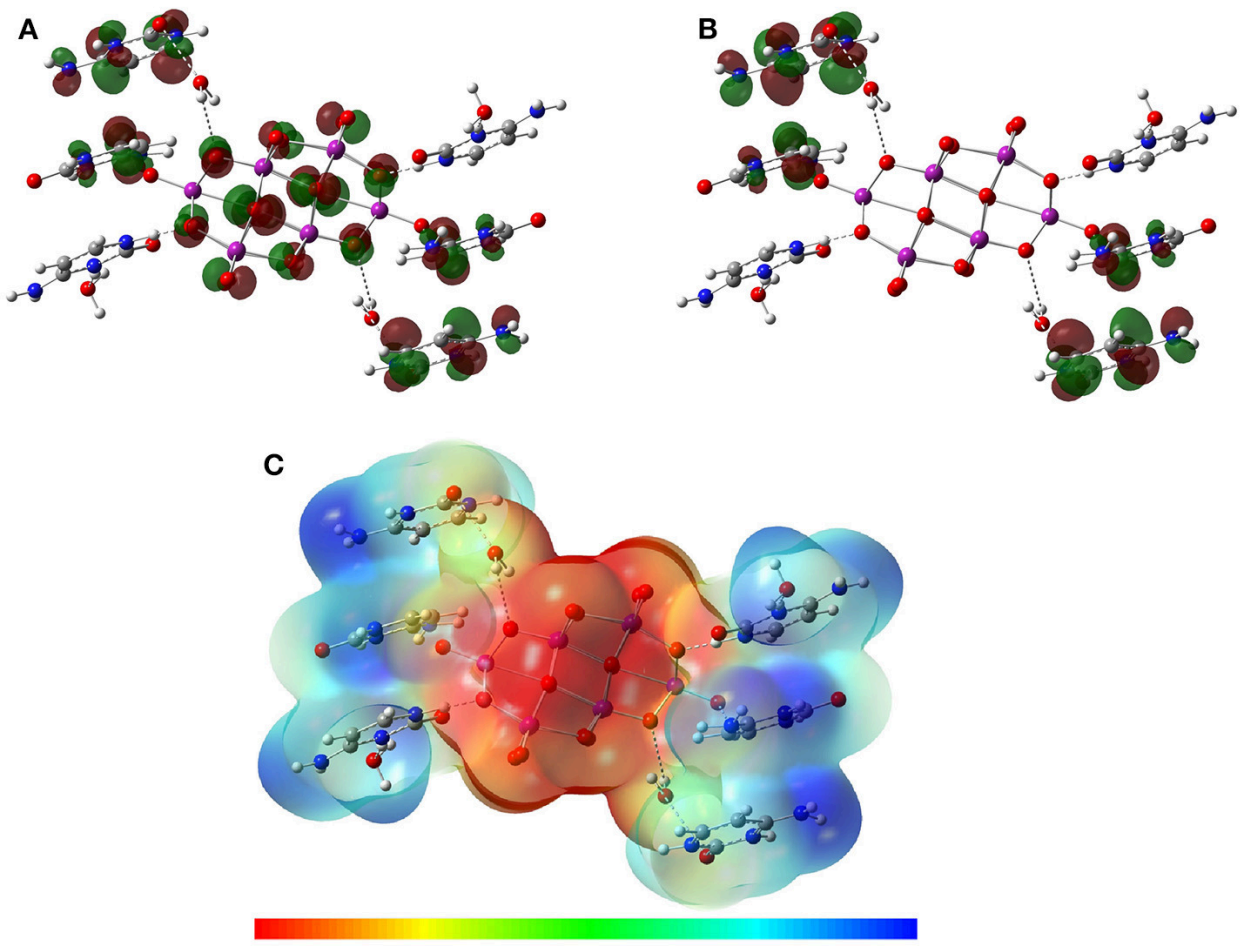
Isosurfaces of frontier molecular orbitals **(A)** HOMO and **(B)** LUMO, and **(C)** MEP of Compound **1** at B97-D3 functional with the 6-31G(d) basis set for C, H, O, N, atoms and LanL2MB basis set for V atoms in solution phase.

**Figure 11:**
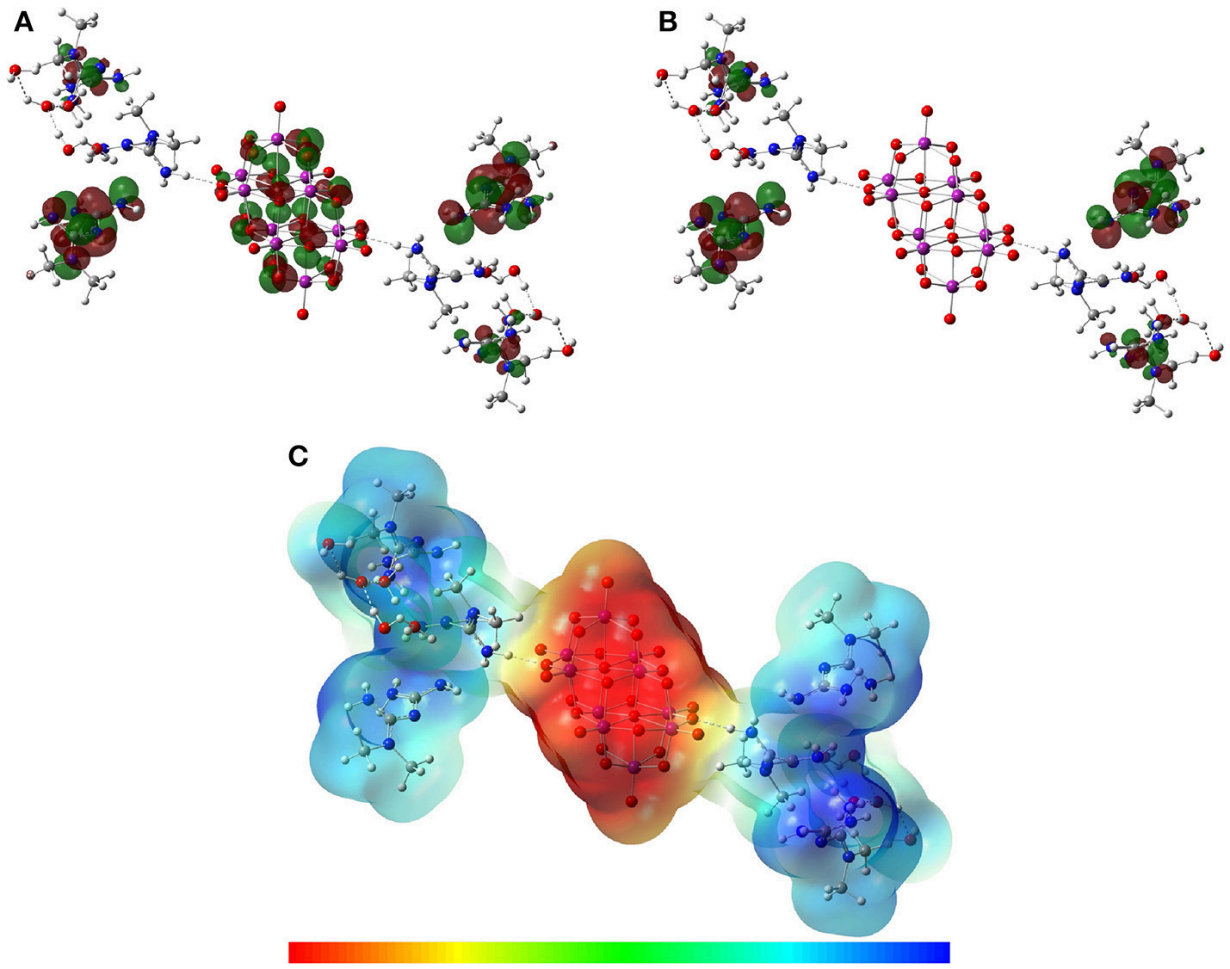
Isosurfaces of frontier molecular orbitals **(A)** HOMO and **(B)** LUMO, and **(C)** MEP of Compound **2** at B97-D3 functional with the 6-31G(d) basis set for C, H, O, N, atoms and LanL2MB basis set for V atoms in solution phase.

Figures 10, 11 show the distribution for the frontier molecular orbitals [the highest occupied molecular orbital (HOMO) and the lowest unoccupied molecular orbital (LUMO)], and the molecular electrostatic potential (MEP) for Compounds **1** and **2**. When the decavanadate anion [V_10_O_28_]^6−^ is built together with cytosinium or metforminium cations and water molecules, significant symmetric non-covalent interactions are observed in the resulting compounds. For compound **1**, the HOMO was mainly delocalized on all oxygen atoms of [V_10_O_28_]^6−^ except O1, as shown in Figure 8. Furthermore, the nitrogen atoms and the partially delocalized C = C double bonds of Cytosinium cations provide a significant contribution to HOMO. On the other hand, LUMO was mainly delocalized on the nitrogen and carbon atoms of cations [C_4_H_6_N_3_O]^+^, (see Figures 10A,B). For compound **2** the HOMO distribution is mainly delocalized on the oxygen atoms of [V_10_O_28_]^6−^, with contributions from the O1, O3 and O5 atoms (and their symmetric equivalents in decavanadate anion), see Figure 9. Additionally, a significant contribution to HOMO is provided from the atoms forming C–N bonds in Metforminium cations. LUMO is mainly delocalized on nitrogen atoms of cations [C_4_H_12_N_5_]^+^ (see Figures 11A,B). The total electron density was mapped with the electrostatic potential surface (isovalue = 0.004) for both compounds, as shown in Figures 10C, 11C. The qualitative color code indicates red regions with a negative charge, while blue regions indicate positive charge. Yellow and green regions correspond to intermediate values toward negative or positive charges, respectively. The negative charge is gathered mainly on the anion [V_10_O_28_]^6−^, while counterions [C_4_H_6_N_3_O]^+^, and [C_4_H_12_N_5_]^+^ have a positive charge. Non-covalent interactions N–H···O and O–H···O are located in intermediate regions of electron density. The distribution of charges in both compounds clearly indicate the relevance of Coulombic interactions.

## Conclusions

This paper focused on the structural description and characterization of two new decavanadate salts, obtained at acidic pH under mild conditions by a combination of ammonium and sodium metavanadates with pharmacologically active counterions from Cytosine and Metformin. In summary, the preparation and complete characterization by elemental analysis, XRD single crystal analysis, spectroscopy and thermal analysis of these new compounds was reported. Also, theoretical studies were carried out to analyze the molecular structure and characterize the intermolecular interactions of these hybrid compounds.

About the crystal structures, we observed that molecular packing in these compounds is predominantly driven by strong hydrogen bonds, as well as Coulombic interactions between cations and decavanadate cluster, building interesting supramolecular networks that have been described herein. The solid-state spectroscopy analyses (FT-IR and Raman) confirmed the presence of [V_10_O_28_]^6−^ and the characteristic vibrations of the counterions. The terminal V = O bonds occur in the 900–1,000 cm^−1^ region; bridging V–O–V bonds vibrate in the 500–700 cm^−1^ region as symmetric and asymmetric stretching modes, respectively, while the V–O bending modes occur in the 300–400 cm^−1^ region. The ^51^V-NMR experiments showed that the equilibrium between decavanadate and vanadate species is progressively displaced toward the formation of V1, V2, and V4 species.

On the other hand, theoretical characterization of optimized structures, frontier molecular orbitals, and the electrostatic potential distribution were obtained. The main non-covalent interactions N–H···O and O–H···O were characterized using DFT-D methods, including dispersion forces that are essential for the treatment of this kind of complexes. The inclusion of solvent effect showed interesting results for correlating the electronic and electrostatic properties with the optimized structures, regardless of those obtained for the crystalline environment.

Currently, further calculations of the theoretical spectroscopic characterization of the IR, Raman, and ^51^V-NMR spectra, as well as a topological analysis of the complexes, are being carried out. Although the scope of this work is far from a biological analysis, we consider that the reported structures can provide interesting insights into their potential pharmacological effects, due to biological activities of the counterions used. First, Compound **2** combined with Metformin shows higher water solubility, becoming promising candidates for carrying out biological studies. Second, it has been reported that decavanadate has anticancer and antidiabetic properties and Metformin, widely recognized as one of the safest and most effective therapeutic compounds for the treatment of Type 2 *D. mellitus* is currently used in the treatment of different types of cancer. Also, deoxycytidine, cytosine arabinoside, and gemcitabine.6 which are derivatives of Cytosine are widely used for the treatment of various malignancies. Benchmark drugs are cytarabine for acute myeloid leukemia and gemcitabine for pancreatic and lung cancer (Muggia et al., 2012).

Biological studies are currently under investigation in our laboratory. However, preliminary results in a mouse colon adenocarcinoma model have shown similar IC50 value as CISPLATIN for Cytosinium decavanadate (Sánchez-Lara et al., 2016a). As for the Metforminium decavanadate 3:1, promising results have been obtained in type 1 and 2 murine models of *D. mellitus*. (Treviño et al., 2015, 2016, 2018). The combination of two therapeutic agents opens up a window toward the generation of potential metalopharmaceuticals with new and exciting pharmacological properties. The results of those investigations will be reported elsewhere in due course.

## Author contributions

All authors listed have made a substantial, direct and intellectual contribution to the work, and approved it for publication.

### Conflict of interest statement

The authors declare that the research was conducted in the absence of any commercial or financial relationships that could be construed as a potential conflict of interest.
