# Supplementary material for: Decavanadate Salts of Cytosine and Metformin: A Combined Experimental-Theoretical Study of Potential Metallodrugs Against Diabetes and Cancer
Source: Front Chem. 2018 Oct 2;6:402. doi: 10.3389/fchem.2018.00402 (PMC6176007; doi:10.3389/fchem.2018.00402)
Supplement: Supplementary file 1 [file Table_1.docx]

Supplementary Material

**Decavanadate Salts of Cytosine and Metformin: A Combined Experimental-Theoretical Study of Potential Metallodrugs Against Diabetes and Cancer**

**Eduardo Sánchez-Lara^1^, Samuel Treviño^2^, Brenda L. Sánchez-Gaytán^1^, Enrique Sánchez-Mora^3^, Maria Eugenia Castro^2^, Francisco Meléndez-Bustamante^2^, Miguel Ángel Mendez-Rojas^4^ and Enrique González-Vergara^1^***

^1^Centro de Química del Instituto de Ciencias, Benemérita Universidad Autónoma de Puebla. 18 Sur y Av. San Claudio. Col. San Manuel. C. P. 72570. Puebla, Pue. Mexico.

^2^Facultad de Ciencias Químicas, Benemérita Universidad Autónoma de Puebla. 18 sur y Av. San Claudio. Col. San Manuel. C. P. 72570. Puebla, Pue. Mexico.

^3^Instituto de Física “Luis Rivera Terrazas”, Benemérita Universidad Autónoma de Puebla. Apdo. Postal J-48, C. P. 72570. Puebla, Pue. Mexico.

^4^Departamento de Ciencias Químico-Biológicas, Universidad de las Américas Puebla, Ex-Hda. Sta. Catarina Mártir, San Andrés Cholula, 72820 Puebla, Mexico

*** Correspondence:**Corresponding Author

Dr. Enrique González-Vergara
[enrique.gonzalez@correo.buap.mx](mailto:enrique.gonzalez@correo.buap.mx)

**Keywords: Decavanadate, Metformin, Cytosine, X-ray crystal structure, Vibrational spectroscopy, ^51^V-NMR, theoretical studies, polyoxovanadates**


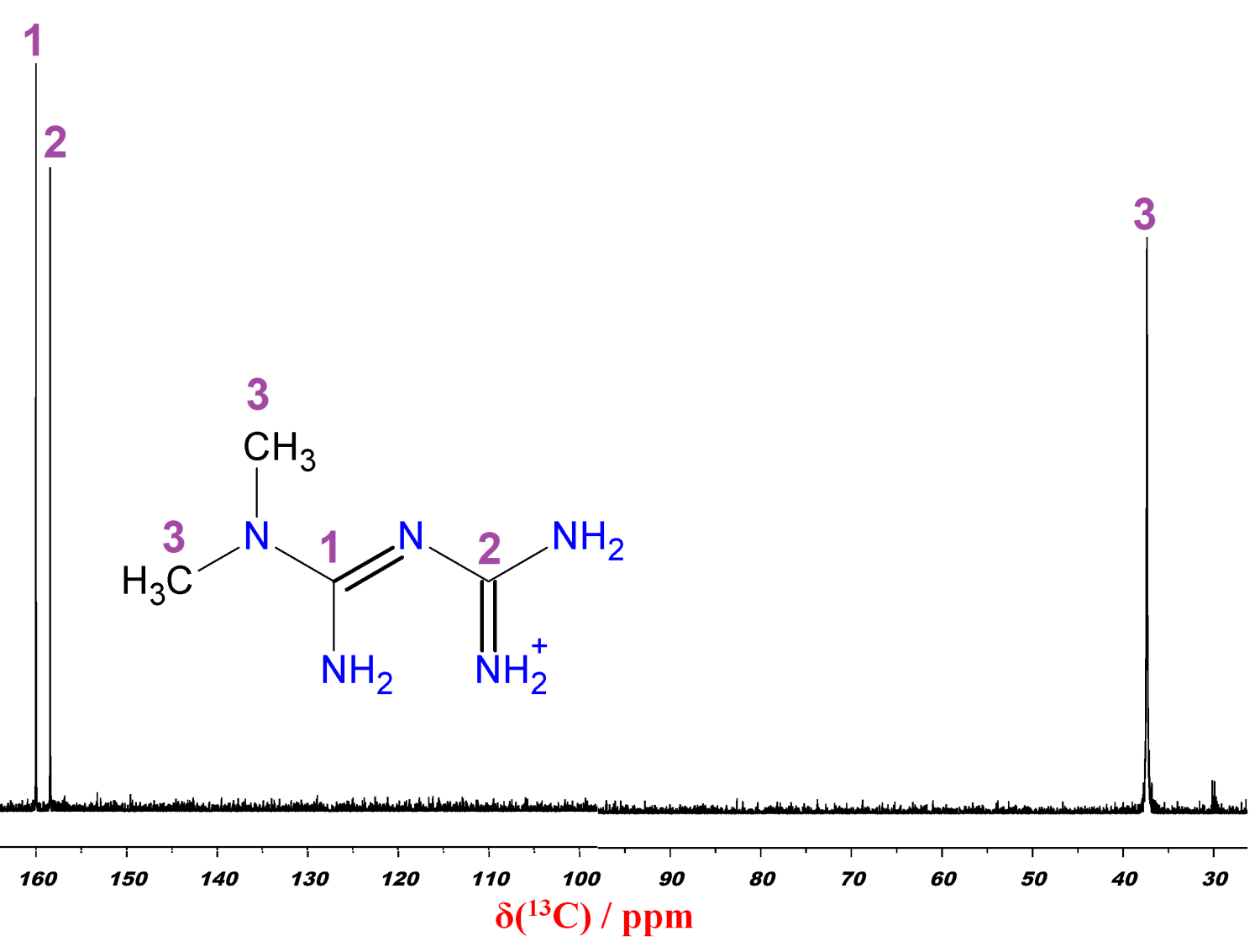


**Supplementary Figure S1**. ^13^C-NMR spectra of Compound **2** in D_2_O.


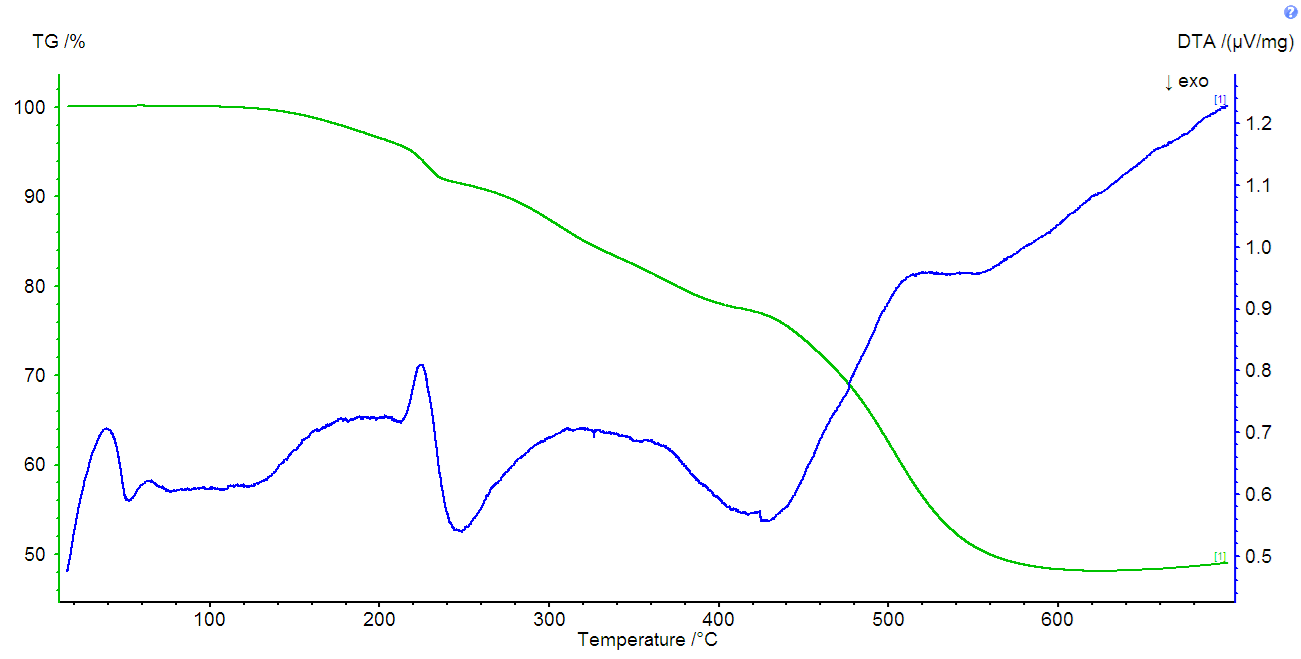


**Supplementary Figure S2.** TGA/DTA for compound **1**


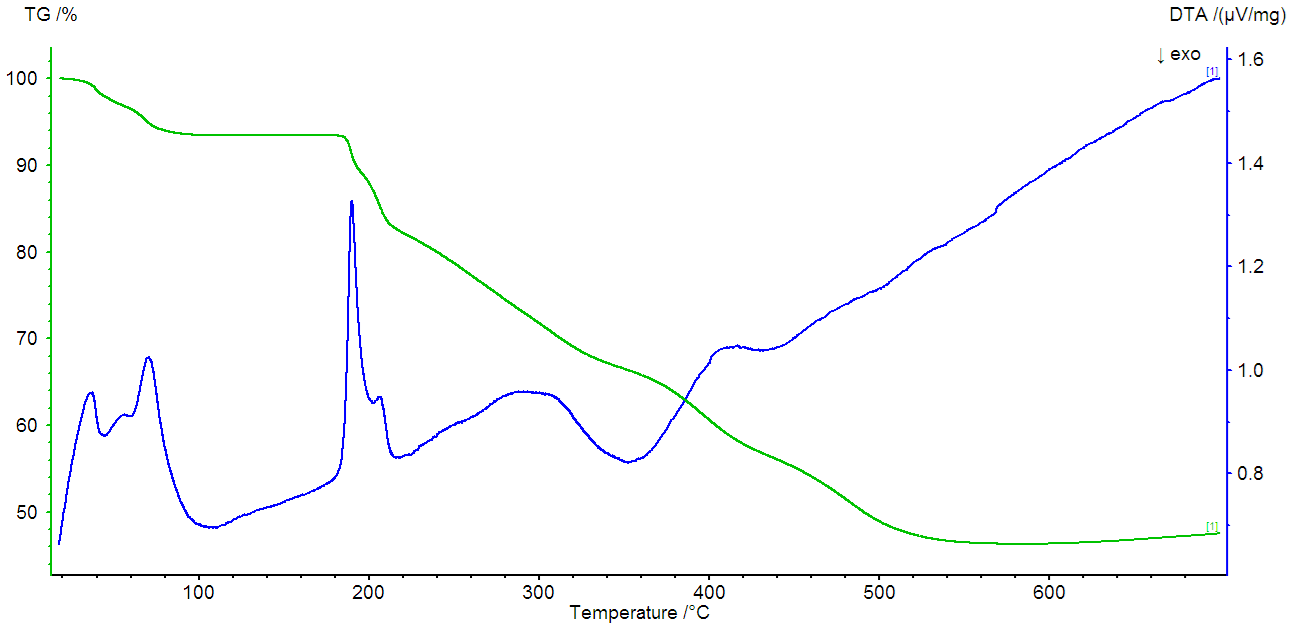


**Supplementary Figure S3.** TGA/DTA for compound **2**

**Supplementary Table S1.** Calculated selected parameters for **1** in aqueous solvation phase. Bond lengths in (Å), bond and dihedral angles in (°).

| Parameter | X-ray data | Solution phase |
| --- | --- | --- |
| V1 O1 | 1.704 | 1.656 |
| V1 O7 | 1.692 | 1.752 |
| V1 O14 | 2.097 | 2.072 |
| V1 O6 | 1.918 | 1.859 |
| V3 O3 | 1.609 | 1.630 |
| C1 O1A | 1.211 | 1.222 |
| C1 N1 | 1.365 | 1.386 |
| C1 N2 | 1.379 | 1.408 |
| C2 N3 | 1.303 | 1.330 |
| C2 N2 | 1.351 | 1.367 |
| O1 V1 O7 | 106.65 | 108.00 |
| O1 V1 O6 | 97.21 | 97.39 |
| O6 V1 O8 | 155.21 | 140.12 |
| O7 V1 O14 | 88.21 | 85.46 |
| N1 C1 O1A | 124.05 | 125.17 |
| N1 C1 N2 | 113.94 | 113.25 |
| N2 C2 N3 | 117.13 | 119.43 |
| C4 N1 C1 | 123.05 | 123.40 |
| H31A O31 H31B | 104.57 | 102.11 |
| H32A O32 H32B | 105.34 | 104.86 |
| O1 V1 O7 O8 | 99.10 | 105.19 |
| O1 V1 O14 V2 | -171.57 | -138.75 |
| O1 V1 O7 O14 | 179.98 | -178.15 |
| O2 V2 O11 V5 | 174.54 | 170.32 |
| O7 V1 O14 V4 | -1.21 | 1.38 |
| O8 V1 O1 O14 | -80.21 | -77.93 |
| C4 N1 C1 O1A | -176.56 | -179.08 |
| O1A C1 N1 N2 | -179.69 | -179.87 |
| C3 C2 N2 N3 | 179.10 | 179.30 |
| C4 N1 C1 N2 | 3.13 | 0.78 |

**Supplementary Table S2.** Calculated selected parameters for **2** in aqueous solvation phase. Bond lengths in (Å), bond and dihedral angles in (°).

| Parameter | X-ray data | Solution phase |
| --- | --- | --- |
| V1 O1 | 1.607 | 1.648 |
| V1 O7 | 1.832 | 1.756 |
| V1 O14 | 2.295 | 1.952 |
| V1 O6 | 1.877 | 1.893 |
| V3 O3 | 1.618 | 1.632 |
| C12 N10 | 1.317 | 1.368 |
| C12 N13 | 1.340 | 1.340 |
| C14 N13 | 1.323 | 1.328 |
| C14 N15 | 1.350 | 1.344 |
| C17 N16 | 1.452 | 1.465 |
| O1 V1 O7 | 103.68 | 111.18 |
| O1 V1 O6 | 100.79 | 93.56 |
| O6 V1 O8 | 154.59 | 145.69 |
| O7 V1 O14 | 82.71 | 88.55 |
| N10 C12 N11 | 118.22 | 117.92 |
| C12 N13 C14 | 122.39 | 125.90 |
| N15 C14 N16 | 118.49 | 117.95 |
| C17 N16 C18 | 115.53 | 115.71 |
| H51A O51 H51B | 104.11 | 105.50 |
| H52A O52 H52B | 104.05 | 103.22 |
| O1 V1 O7 O8 | 103.75 | 100.99 |
| O1 V1 O14 V2 | 170.58 | 123.01 |
| O1 V1 O7 O14 | -178.91 | -179.19 |
| O2 V2 O11 V5 | 179.14 | 172.56 |
| O7 V1 O14 V4 | -0.76 | -0.84 |
| O8 V1 O1 O14 | -94.56 | -76.51 |
| N10 C12 N11 N13 | -177.48 | -178.20 |
| C12 N13 C14 N15 | 26.22 | 12.96 |
| N15 C14 N16 C17 | 2.00 | 13.69 |
| C14 N16 C17 C18 | 175.94 | 177.95 |

**Supplementary Table S3.** Principal calculated hydrogen bonds (Å, °) for **1** and **2** in aqueous solvation phase.

| *D*—H···*A* | *D*—H | H···*A* | *D*···*A* | *D*—H···*A* |
| --- | --- | --- | --- | --- |
| Compound **(1)** | | | | |
| N11—H11···O12 | 1.050 | 1.656 | 2.698 | 171.32 |
| N12—H12···O32 | 1.045 | 1.744 | 2.735 | 156.59 |
| O31—H31A···O7 | 0.981 | 2.565 | 2.973 | 104.89 |
| O31—H31B···O1A | 0.976 | 2.145 | 3.032 | 150.33 |
| Compound **(2)** | | | | |
| N35A—H35*A*···O8 | 1.031 | 1.910 | 2.903 | 160.38 |
| O52$-$H52A⋅⋅⋅O54 | 0.985 | 1.877 | 2.861 | 177.14 |
| O53$-$H53A⋅⋅⋅O54 | 0.996 | 1.769 | 2.749 | 167.148 |
