# Supplementary figures and images for: Decavanadate Salts of Cytosine and Metformin: A Combined Experimental-Theoretical Study of Potential Metallodrugs Against Diabetes and Cancer
Source: Front Chem. 2018 Oct 2;6:402. doi: 10.3389/fchem.2018.00402 (PMC6176007; doi:10.3389/fchem.2018.00402)

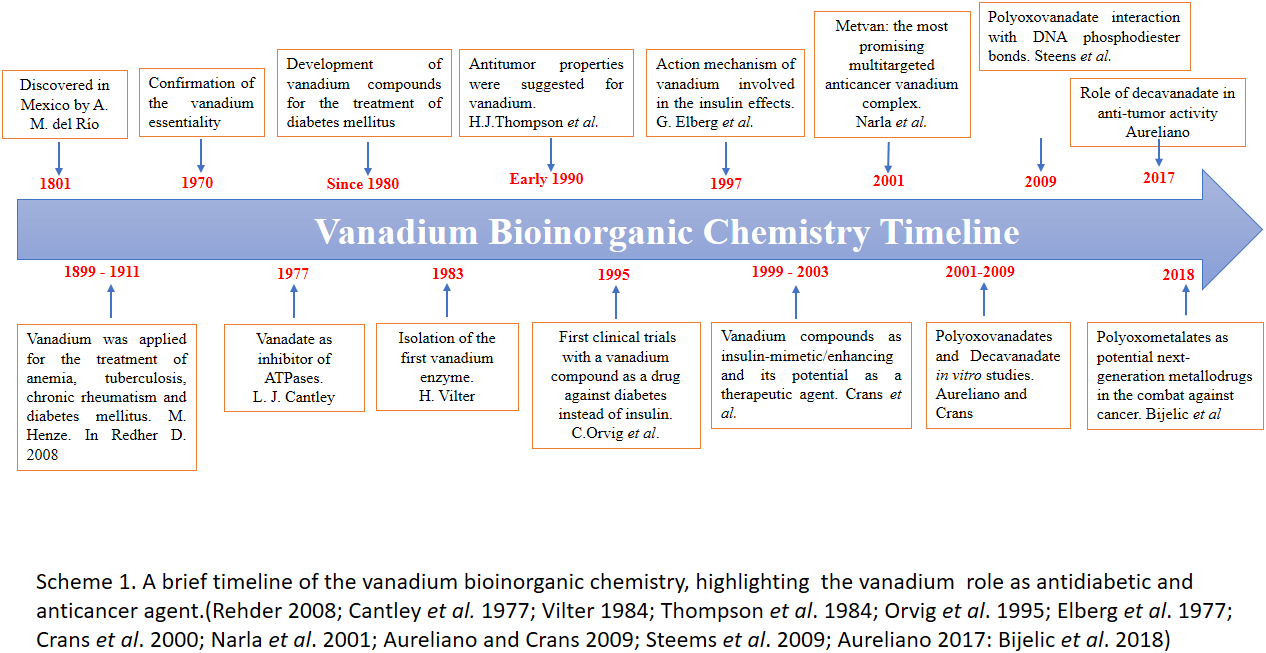

Supplement: Supplementary file 2 [file Image_1.TIF]

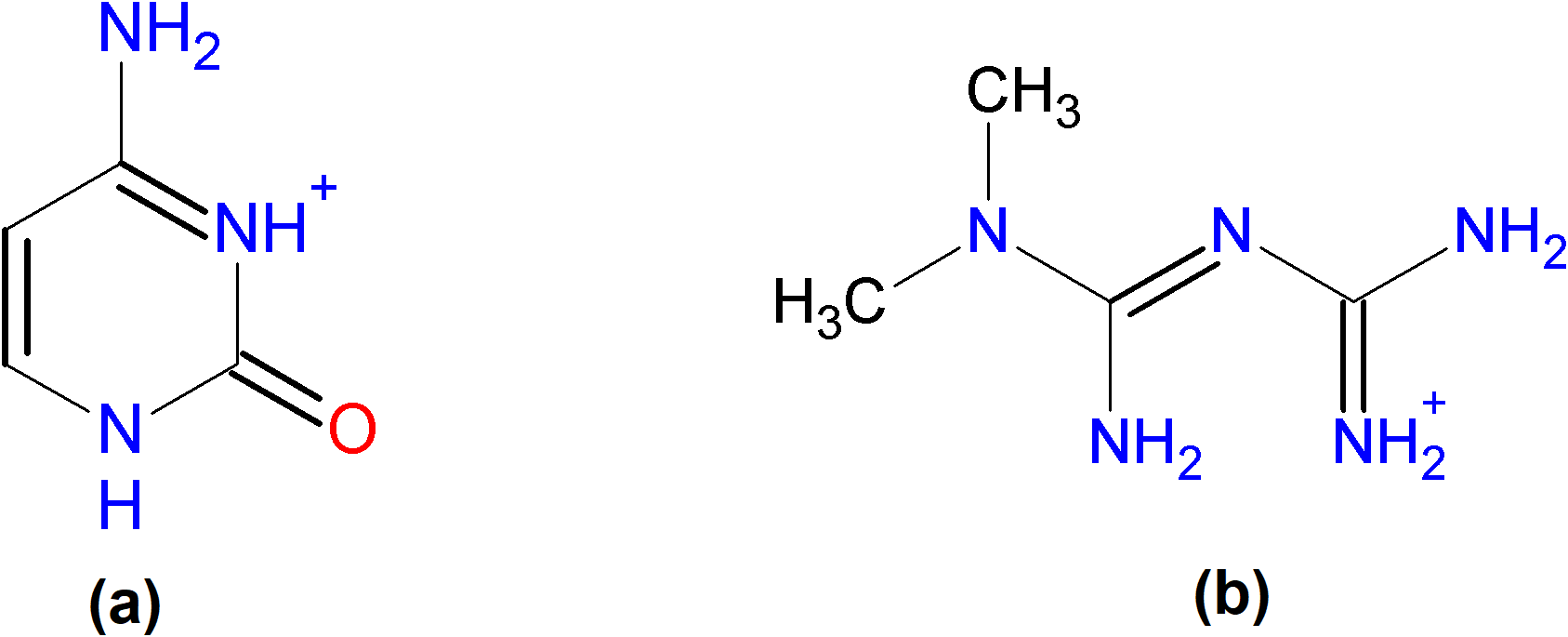

Supplement: Supplementary file 3 [file Image_2.TIF]

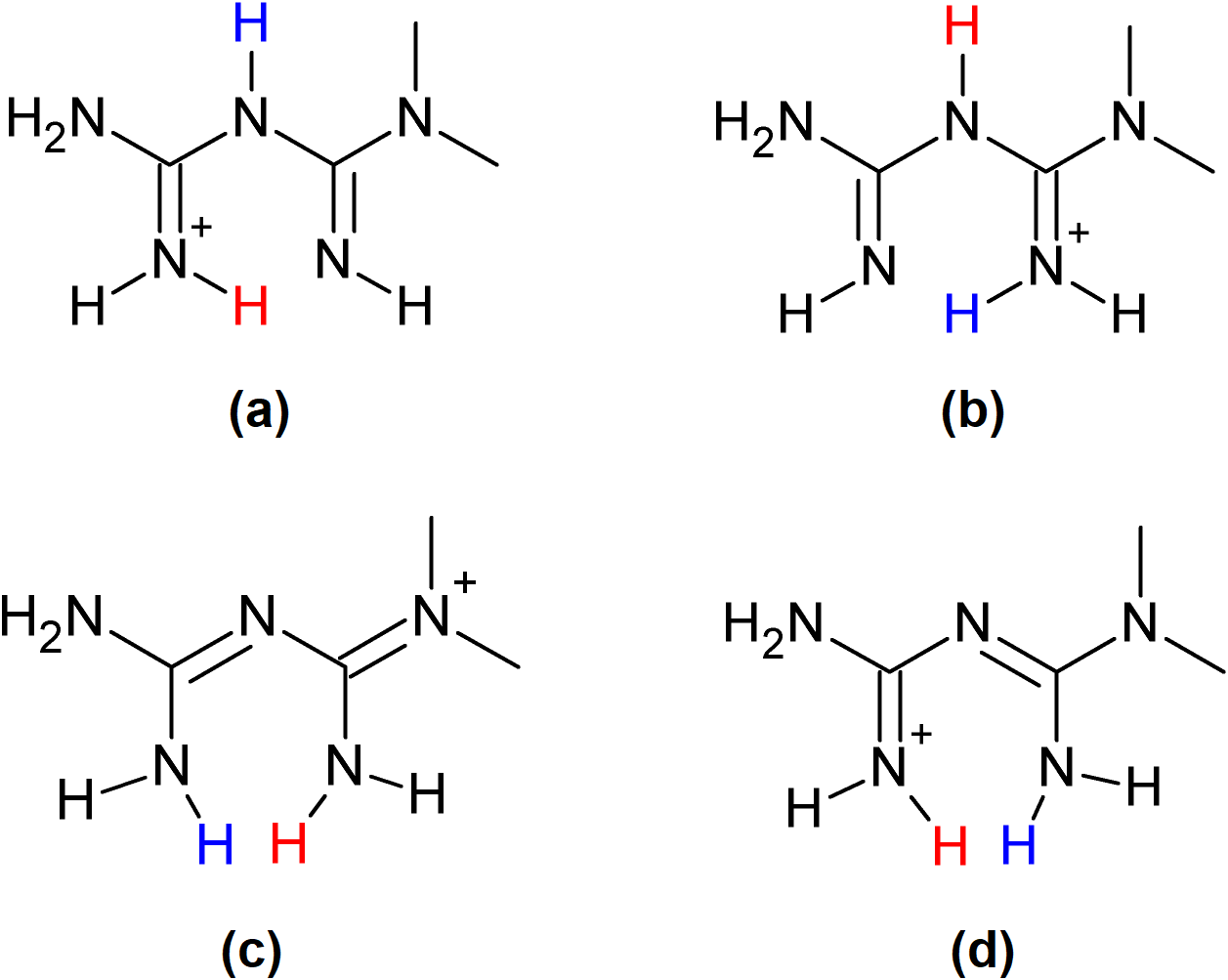

Supplement: Supplementary file 4 [file Image_3.TIF]
